# Deciphering the Routes of invasion of *Drosophila suzukii* by Means of ABC Random Forest

**DOI:** 10.1093/molbev/msx050

**Published:** 2017-02-25

**Authors:** Antoine Fraimout, Vincent Debat, Simon Fellous, Ruth A. Hufbauer, Julien Foucaud, Pierre Pudlo, Jean-Michel Marin, Donald K. Price, Julien Cattel, Xiao Chen, Marindia Deprá, Pierre François Duyck, Christelle Guedot, Marc Kenis, Masahito T. Kimura, Gregory Loeb, Anne Loiseau, Isabel Martinez-Sañudo, Marta Pascual, Maxi Polihronakis Richmond, Peter Shearer, Nadia Singh, Koichiro Tamura, Anne Xuéreb, Jinping Zhang, Arnaud Estoup

**Affiliations:** 1 Institut de Systématique, Évolution, Biodiversité, ISYEB - UMR 7205 – CNRS, MNHN, UPMC, EPHE, Muséum national d’Histoire naturelle, Sorbonne Universités, Paris, France; 2 INRA, Centre de Biologie et de Gestion des Populations (UMR INRA IRD Cirad Montpellier SupAgro), Montferrier-Sur-Lez, France; 3 Colorado State University, Fort Collins, CO; 4 Centre de Mathématiques et Informatique, Aix-Marseille Université, Marseille, France; 5 Institut Montpelliérain Alexander Grothendieck, Université de Montpellier, Montpellier, France; 6 Tropical Conservation Biology & Environmental Science, University of Hawaii at Hilo, HI; 7 Laboratoire de Biométrie et Biologie Evolutive, UMR CNRS 5558, Université Claude Bernard Lyon 1, Villeurbanne, France; 8 College of Plant Protection, Yunnan Agricultural University, Kunming, Yunnan Province, People’s Republic of China; 9 Programa de Pós Graduação em Genética e Biologia Molecular, Programa de Pós Graduação em Biologia Animal, Universidade Federal do Rio Grande do Sul, Porto Alegre, Brazil; 10 CIRAD, UMR Peuplement Végétaux et Bioagresseurs en Milieu Tropical, Paris, France; 11 Department of Entomology, University of Wisconsin, Madison, WI; 12 CABI, Delémont, Switzerland; 13 Graduate School of Environmental Earth Science, Hokkaido Daigaku University, Sapporo, Hokkaido Prefecture, Japan; 14 Department of Entomology, Cornell University, Ithaca, NY; 15 Dipartimento di Agronomia Animali Alimenti Risorse Naturali e Ambiente, Universita degli Studi di Padova, Padova, Italy; 16 Departament de Genètica, Universitat de Barcelona, Barcelona, Spain; 17 Division of Biological Sciences, University of California San Diego; 18 Mid-Columbia Agricultural Research and Extension Center, Oregon State University, Hood River, OR; 19 Department of Genetics, North Carolina State University, Raleigh, NC; 20 Department of Biological Sciences, Tokyo Metropolitan University, Tokyo, Japan; 21 MoA-CABI Joint Laboratory for Bio-safety, Chinese Academy of Agricultural Sciences, BeiXiaGuan, Haidian Qu, China

**Keywords:** *Drosophila suzukii*, invasion routes, random forest, approximate Bayesian computation, population genetics

## Abstract

Deciphering invasion routes from molecular data is crucial to understanding biological invasions, including identifying bottlenecks in population size and admixture among distinct populations. Here, we unravel the invasion routes of the invasive pest *Drosophila suzukii* using a multi-locus microsatellite dataset (25 loci on 23 worldwide sampling locations). To do this, we use approximate Bayesian computation (ABC), which has improved the reconstruction of invasion routes, but can be computationally expensive. We use our study to illustrate the use of a new, more efficient, ABC method, ABC random forest (ABC-RF) and compare it to a standard ABC method (ABC-LDA). We find that Japan emerges as the most probable source of the earliest recorded invasion into Hawaii. Southeast China and Hawaii together are the most probable sources of populations in western North America, which then in turn served as sources for those in eastern North America. European populations are genetically more homogeneous than North American populations, and their most probable source is northeast China, with evidence of limited gene flow from the eastern US as well. All introduced populations passed through bottlenecks, and analyses reveal five distinct admixture events. These findings can inform hypotheses concerning how this species evolved between different and independent source and invasive populations. Methodological comparisons indicate that ABC-RF and ABC-LDA show concordant results if ABC-LDA is based on a large number of simulated datasets but that ABC-RF out-performs ABC-LDA when using a comparable and more manageable number of simulated datasets, especially when analyzing complex introduction scenarios.

## Introduction

Biological invasions are a component of global change, and their impact on the communities and ecosystems they invade is substantial (Simberloff 2013). Invasive populations evolve rapidly, via both neutral and selective evolutionary processes, as they undergo dramatic range expansion, demographic reshuffling and experience new selection regimes (Keller and Taylor 2010; Dlugosch et al. 2015). While extensive literature has laid the foundations of an evolutionary framework of biological invasions (Sakai et al. 2001; Lee 2002; Facon et al. 2006; Dlugosch et al. 2015; Estoup et al. 2016), whether evolutionary processes are drivers of invasion success or outcomes of introduction processes remains unclear.

Two of the evolutionary processes hypothesized to play a role in invasions are demographic bottlenecks and genetic admixture (Ellstrand and Shierenbeck 2000; Dlugosch and Parker 2008; Rius and Darling 2014). Demographic bottlenecks, which correspond to transitory reductions in population size associated with invasions, can reduce genetic variation and thus may constrain invasion success (Edmonds et al. 2004; Dlugosch and Parker 2008; Peischl and Excoffier 2015). A diverse array of mechanisms allows bottlenecked populations to overcome the deleterious consequences of low genetic variation and adapt to their novel environments [reviewed in Estoup et al. (2016)]. Genetic admixture occurs when multiple introduction events derive from genetically differentiated native or invasive populations. Admixure can increase genetic diversity in introduced populations, potentially enhancing invasion success by increasing genetic variation on which selection can act (Kolbe et al. 2004; Lavergne and Molofsky 2007) or via producing entirely novel genotypes that may facilitate colonization of novel habitats (Dlugosch and Parker 2008; Rius and Darling 2014). It remains unclear, however, how often genetic admixture occurs in invasions and whether it acts as a true driver of invasion success (Uller and Leimu 2011; Rius and Darling 2014).

To evaluate hypotheses regarding bottlenecks and admixture, as well as adaptation or other processes that may occur during and after introductions, we must first identify the original native or invasive source(s) of the introductions. By comparing introduced to likely source populations, one can infer whether populations diverged during invasion and then start to distinguish among evolutionary processes that drive observed differences (Dlugosch and Parker 2008; Keller and Taylor 2010). Typically, accurate historical data on introductions pathways are limited (Estoup and Guillemaud 2010). Traditional population genetic approaches can describe the genetic structure and relationships among sampled native and invasive populations (Boissin et al. 2012), but determining origins of introduced species from population genetic data is challenging. This challenge prompted the development of statistical methods such as approximate Bayesian computation (ABC; Beaumont et al. 2002). In ABC analysis, different introduction scenarios that describe possible introduction pathways are compared quantitatively, taking a model-based Bayesian approach. Datasets matching different scenarios are generated by simulation, and by comparing these simulated datasets to the observed dataset, approximate posterior probabilities of the scenarios are estimated (Bertorelle et al. 2010; Beaumont 2010). A crucial step in determining the most probable introduction pathway is to formally describe a finite set of possible introduction scenarios as models (Estoup and Guillemaud 2010). These introduction pathways should be based on temporal/historical information about invasions where feasible, and they can be realistically complex. For example, rather than a species moving directly from the native to invasive range, it might first pass through another region, experiencing bottlenecks and/or admixture along the way. ABC has been applied successfully to a wide range of case studies, from pest invasion (e.g., *Harmonia axiridis*, Lombaert et al. 2014) to anthropological research (e.g., *Homo sapiens*, Verdu et al. 2009) and is now widely used by the population genetics community. However, and despite the many advantages of ABC, it relies on massive simulations that can render it computationally costly. When multiple complex introduction scenarios are considered, the time and computational resources required to choose among them can become prohibitive (Lombaert et al. 2014). To overcome this constraint, a new algorithm called ABC random forest (ABC-RF) has been developed (Pudlo et al. 2016). Simulations show that ABC-RF discriminates among scenarios more efficiently than traditional ABC methods (Pudlo et al. 2016), and thus it appears particularly suitable for distinguishing among complex invasion pathways (see section below *New approaches*).

Here, we evaluate the origins of invasive populations of spotted-wing *Drosophilla suzukii* (Matsumura 1931), including where and whether they passed through bottlenecks in populations size, or experienced admixture between genetically distinct groups. We use this invasion to illustrate the use of ABC-RF, and as a case study to compare it to a more standard ABC method. *Drosophila suzukii* is a representative of the *melanogaster* group and is historically distributed in Southeast Asia, covering a large portion of China, Japan, Thailand and neighboring countries (Asplen et al. 2015). Unlike most drosophilid species, *D. suzukii* females display a large serrated ovipositor (Atallah et al. 2014) allowing them to lay eggs in ripening fruits, and they are thus a major threat to the agricultural production of stone fruits and berries (Lee et al. 2011). The presence of the species outside of its native range was first recorded in the Hawaiian archipelago in the early 1980's (Kaneshiro 1983) but no further spread was observed until the late 2000's when *D. suzukii* was almost synchronously recorded in the southwest of the USA and southern Europe (Hauser 2011; Calabria et al*.* 2012). By 2015, the species had spread throughout most of the North-American and European continents and reached the southern hemisphere in Brazil (Deprá et al. 2014). *Drosophila suzukii* represents a particularly appealing biological model for gaining further insights into the evolutionary factors associated with invasion success. As a closely related species of the model insect *Drosophila melanogaster*, its short generation time and viability in laboratory conditions facilitate experimental approaches to study evolutionary events.

ABC-based analyses of DNA sequences data obtained for six X-linked gene fragments suggest that North American and European populations represent separate invasion events (Adrion et al. 2014). The sources of these invasions and potential admixture among different regions remain unclear. The wide native and introduced distribution of *D. suzukii* imply a potentially large number of possible sources and hence a large number of possible introduction scenarios. By first grouping sample size into genetic clusters, the number of populations treated independently can be reduced somewhat (Lombaert et al. 2014), but not fully. Additionally, the temporal proximity of invasions in the US and Europe makes it difficult to a priori propose certain introduction scenarios over others. Together, these features of *D. suzukii’s* invasion mean that a large number of scenarios will need to be compared, dramatically increasing computational requirements for traditional ABC methods, and hence making ABC-RF methods particularly appealing.

### New Approaches

The formal description of a finite set of possible introduction scenarios as models lays the foundation for ABC analyses, and is outlined in Estoup and Guillemaud (2010). Choosing among the formulated models is the central statistical problem to be overcome when reconstructing routes of invasion from molecular data. Both theoretical arguments and simulation experiments indicate that approximate posterior probabilities estimated from ABC analyses for the modeled introduction scenarios can be inaccurate, even though the models being compared can still be ranked appropriately using numerical approximation (Robert et al. 2011). To overcome this problem, Pudlo et al. (2016) developed a novel approach based on a machine learning tool named “random forests” (RF; Breiman 2001), which selects among the complex introduction models covered by ABC algorithms. This approach enables efficient discrimination among models and estimation of posterior probability of the best model while being computationally less intensive.

We invite readers to consult Pudlo et al. (2016) to access to detailed statistical descriptions and testing of the ABC random forest (ABC-RF) method. Briefly, random forest (RF) is an algorithm that learns from a database how to predict a variable called the output from a possibly large set of covariates. In our context, the database is the reference table which includes a given number of datasets that have been simulated for different scenarios using parameter values drawn from prior distributions, each dataset being summarized with a pool of statistics (i.e., the covariates). RF aggregates the predictions of a collection of classification or regression trees (depending whether the output is categorical, here the scenario identity, or quantitative, here the posterior probability of the best scenario). Each tree is built using the information provided by a bootstrap sample of the database and manages to capture one part of the dependency between the output and the covariates. Based on these trees which are separately poor to predict the output, an ensemble learning technique such as RF aggregates their predictions to increase predictive performances to a high level of accuracy in favorable contexts (Breiman 2001). RF is currently considered as one of the major state-of-art algorithm for classification or regression.

In ABC random forest (ABC-RF), Pudlo et al. (2016) makes two important advances regarding the use of summary statistics, and the identification of the most probable model. For the summary statistics, given a pool of different metrics available, ABC-RF extracts the maximum of information from the entire set of proposed statistics. This avoids the arbitrary choice of a subset of statistics, which is often applied in ABC analyses, and also avoids what in the statistical sciences is called “the curse of dimensionality” [see Blum *et al.* (2013) for a comparative review of dimension reduction methods in ABC]. With respect to identifying the most probable model, ABC-RF uses a classification vote system rather than the posterior probabilities traditionally used in ABC analysis. The first outcome of a ABC-RF analysis applied to a given target dataset is hence a classification vote for each competing model, which represents the number of times a model is selected in a forest of *n* classification trees. The model with the highest number of classification votes corresponds to the model best suited to the target dataset among the set of competing models. As a byproduct, this step also provides a measure of the classification error called the prior error rate. The prior error rate is calculated as the probability of choosing a wrong model when drawing model index and parameter values into priors. The second outcome of ABC-RF is an estimation of the posterior probability of the best model that has been selected, using a secondary random forest that regresses the model selection error of the first-step random forest over the available summary statistics.

Pudlo et al. (2016) shows that, as compared to previous ABC methods, ABC-RF offers at least four advantages (i) it significantly reduces the model classification error as measured by the prior error rate; (ii) it is robust to the number and choice of summary statistics, as RF can handle many superfluous and/or strongly correlated statistics with no impact on the performance of the method [see Blum et al. (2013) for alternative methods of dimension reduction]; (iii) the computing effort is considerably reduced as RF requires a much smaller reference table compared with alternative methods (i.e., a few thousands of simulated datasets versus hundreds of thousands to millions of simulations per compared model); and (iv) it provides more reliable estimation of posterior probability of the selected model (i.e., the model that best fit the observed dataset).

To the best of our knowledge, the present study is the first to use the ABC-RF method for an ensemble of model choice analyses with different levels of complexity (i.e., various number of compared models including various and sometimes large number of parameters, sampled populations and hence number of summary statistics) on real multi-locus microsatellite datasets. Specifically, we analyze molecular data at 25 microsatellite loci from 23 *D. suzukii* sample sites located across most of its native and introduced range. We conducted our population genetics study in five steps. (1) We defined focal genetic groups, and used this information to formalize 11 sets of competing introduction scenarios. We then choose among them in a sequential ABC-RF analysis. (2) We compared the performance of ABC-RF to a more standard ABC method for a subset of analyses [ABC-LDA; Estoup *et al.* (2012); see “Materials and Methods” section]. We choose ABC-LDA in our comparative study because it is considered to be one of the most efficient standard ABC methods to discriminate among models (Pudlo et al. 2016) and it is widely used in population genetics studies (Lombaert et al. 2014, and reference therein). (3) We refined the inferred worldwide invasion scenario by assessing the most likely origins of the primary introductions from the native area. (4) We estimated the posterior distributions under the final invasion scenario of demographic parameters associated with bottleneck and genetic admixture events. (5) Finally, we performed model-posterior checking analyses to insure that the final worldwide invasion scenario displayed a reasonable match to the observed dataset.

## Results

### Origins of Invasive Genetic Groups

Prior to conducting any type of ABC analysis, focal genetic groups must be defined by characterizing genetic diversity and structure within and between all genotyped sample sites using traditional statistics and clustering methods. This is described in details in supplementary appendix S1, Supplementary Material online. We defined seven main genetic groups from our initial set of 23 sample sites (supplementary table S1, Supplementary Material online): Asia (sample sites CN-Lan, CN-Lia, CN-Nin, CN-Shi, JP-Tok, and JP-Sap), Hawaii (sample site US-Haw), western US (sample sites US-Wat, US-Sok, and US-SD), eastern US (sample sites US-Col, US-NC, US-Wis, and US-Gen), Europe (sample sites GE-Dos, FR-Par, FR-Bor, FR-Mon, SW-Del, SP-Bar, and IT-Tre), Brazil (BR-PA), and La Réunion (FR-Reu).

This genetic grouping along with historical and geographical information (supplementary table S1, Supplementary Material online) allowed us to formalize 11 nested sets of competing invasion scenarios that we analyzed sequentially using ABC model choice methodologies. The date that *D. suzukii* was first recorded in a location was used to help formulate the scenarios. For example, *D. suzukii* was first recorded in the continental US from California (US-Wat) in 2008 and was not recorded in the central state of Colorado (US-Col) until 2012. US-Wat could therefore serve as a source for US-Col, but not *vice versa*. We present the main analyses in table 1. In supplementary table S2, Supplementary Material online, a more detailed description of each competing scenarios considered for each analysis is provided. The 11 sequential ABC analyses permit step-by-step reconstruction of the introduction history of *D. suzukii* worldwide. Results for each analysis based on prior set 1 (which used bounded uniform distributions of parameters; see “Materials and Methods” section) and a single set of sample sites representative of the genetic groups defined above are given in table 2. For all 11 analyses, similar ABC-RF results were obtained using the prior set 2 (which used more peaked distributions; supplementary table S3, Supplementary Material online) and considering various sets of representative sample sites (supplementary table S4, Supplementary Material online).

**Table 1:** Formulation of the Model Choice Analyses that were Carried Out Successively to Reconstruct Invasion Routes of *D. suzukii* Using ABC.

Model Choice Analysis	Number of Compared Scenarios	Tackled Question	Potential source genetic group	Focal populations
1a	3	What are the origins of western US populations?	Asia, Hawaii	US-Wat
1b				US-Sok
1c				US-SD
1d	7	What are the relations among western US populations and their extra-continental sources?	Asia, Hawaii, US-Wat, US-Sok, US-SD	US-Wat + US-Sok + US-SD
2a	6	What are the origins of eastern US populations?	Asia, Hawaii, western US	eastern US
2b	6	What are the origins of European populations?	Asia, Hawaii, western US	Europe
3a	10	Is there asymmetrical gene flow from Europe to eastern US?	Asia, Hawaii, western US, Europe	eastern US
3b	10	Is there asymmetrical gene flow from eastern US to Europe?	Asia, Hawaii, western US, eastern US	Europe
4	2	Does the admixture with eastern US genes in northern Europe result from a secondary Asian introduction?	Asia, Hawaii, western US, eastern US, southern Europe	northern Europe
5a	21	What are the origins of the Brazilian population?	Asia, Hawaii, western US, eastern US, southern Europe, northern Europe	Brazil
5b	21	What are the origins of La Reunion population?	Asia, Hawaii, western US, eastern US, southern Europe, northern Europe	La Reunion

Note*.*— Each numbered analysis is a comparison of a certain number of scenarios by ABC model choice. We summarize each analysis by stating the question that it addressed. A detailed verbal description of each compared scenario is given in supplementary table S2, Supplementary Material online. The 11 analyses are nested in the sense that each subsequent analysis use the result obtained from the previous one. For example, in Analysis 2a “What are the origins of eastern US populations” capitalizes on the history inferred for western US populations from analysis 1d. “Potential source genetic group” indicates all the potential source populations considered in the analyses for which one wants to identify the origin of the focal population (i.e., the target).

**Table 2:** Results of Model Choice Analyses Using ABC-RF and ABC-LDA.

			Prior error rate			Posterior Probability of the best Model		Origin of the focal population using either ABC-RF or ABC-LDA (i.e., best model)
Analysis	Total Number of Scenarios	Number of Sum. Stats.	ABC-RF (s.d.)	ABC-LDA (large reference table)	ABC-LDA (small reference table)	ABC-RF (s.d.)	ABC-LDA (large reference table) [CI]	
1a	3	39	0.100 (± 0.002)	0.075	0.085	1.000 (± 0.001)	1.000 [1.000, 1.000]	Asia + Hawaii
1b	3		0.094 (± 0.001)	0.070	0.091	0.989 (± 0.005)	0.999 [0.999, 0.999]	
1c	3		0.096 (± 0.001)	0.079	0.087	0.998 (± 0.002)	0.999 [0.999, 0.999]	
1d	7	130	0.327 (± 0.001)	0.274	0.368	0.690 (± 0.018)	0.741 [0.714, 0.767]	Asia + Hawaii
								
2a	6	130	0.232 (± 0.001)	0.203	0.241	0.779 (± 0.019)	0.788 [0.764, 0.813]	Western US
								
2b	6	130	0.232 (± 0.001)	0.179	0.393	0.716 (± 0.013)	0.604 [0.592, 0.616]	Asia
								
3a	10	204	0.328 (± 0.001)	0.265	0.364	0.744 (± 0.020)	0.844 [0.820, 0.868]	Western US
								
3b	10	204	0.408 (± 0.001)	0.358	0.423	0.510 (± 0.014)	0.409 [0.391, 0.426]	Asia
								
4	2	301	0.121 (± 0.001)	0.111	0.212	1.000 (± 0.009)	0.999 [0.999, 0.999]	Southern Europe + eastern US
							
5a	21	424	0.294 (± 0.001)	0.230	0.423	0.631 (± 0.034)	0.812 [0.788, 0.837]	Western US + eastern US
							
5b	21	424	0.298 (± 0.001)	0.242	0.428	0.500 (± 0.027)	0.290 [0.256, 0.323]	Northern Europe + southern Europe
							

**Fig. 1:**
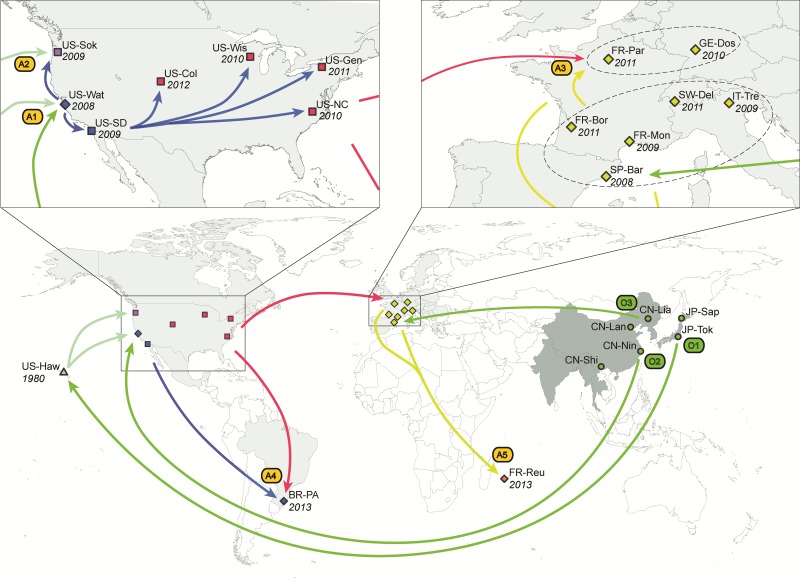
Worldwide invasion scenario of *D. suzukii* inferred from microsatellite data and date of first observation. Map and schematic showing sample sites and the invasion routes taken by *D. suzukii*, as reconstructed by ABC-RF (Pudlo et al. 2016) on a total of 685 individuals from 23 geographic locations genotyped at 25 microsatellite loci (see results and methods for details). The native range is in dark grey, and the invasive range is in light-gray (cf. delimitation from fig. 1 in Asplen et al. 2015). The year in which *D. suzukii* was first observed at each sample site is indicated in italics. The 23 geographical locations that were sampled are represented by circles (native range), and squares, diamonds and triangles (introduced range). Squares indicate populations that experienced weak bottlenecks (i.e., median value of bottleneck severity < 0.12, see main text and table 3), diamonds indicate moderate bottlenecks (0.12 < bottleneck severity < 0.22) and triangles indicate strong bottlenecks (i.e., bottleneck severity > 0.3). The colors of the symbol for the sample sites and arrows between them correspond to the different genetic groups obtained using the clustering method BAPS (supplementary appendix S1, Supplementary Material online). The arrows indicate the most probable invasion pathways. A1–A5 indicate five separate admixture events between different sources. O1–O3 indicate the most probable sources within the native range for the primary introduction events. A1 = Hawaii + southeast China; A2 = Watsonville (western US) + Hawaii; A3 = southern Europe + eastern US; A4 = western US + eastern US; A5 = southern Europe + northern Europe. O1 = Japan; O2 = southeast China; O3 = northeast China.

Note.— All model choice analyses were carried out using the prior set 1 (supplementary table S8, Supplementary Material online) and a single set of sample sites representative of the pre-defined genetic groups. Sample site JP-Tok for the native Asian group, US-Haw for the Hawaiian group, US-Wat, US-Sok and US-SD for the western US group, US-NC for the eastern US group, IT-Tre for the southern Europe group, GE-Dos for the northern Europe group, BR-PA for the Brazil group, and FR-Reu for the La Réunion group (fig. 1). Datasets were summarized using the whole set of summary statistics proposed by DIYABC (Cornuet et al. 2014). The total number of summary statistics (Number of Sum. Stats.) as well as the total number of compared scenarios (Total number of scenarios) are indicated for each analysis. Prior error rates and posterior probabilities of the best model chosen using ABC-RF were averaged over 10 replicate analyses. ABC-RF and ABC-LDA treatments yielded the same best model choice for all analyses and is denoted in the column “Origin of the focal population using either ABC-RF or ABC-LDA (i.e., best model)”. Admixture between two source populations are represented by a “+” sign. ABC-LDA posterior probabilities of the best models were estimated using “large reference tables” with 500,000 simulated datasets per scenario. ABC-LDA prior error rates were computed using reference tables of two different sizes: “large reference table” (i.e., 500,000 simulated datasets per scenario) and “small reference table” (i.e., 10,000 simulated datasets per scenario as for ABC-RF analyses). S.D. stands for standard deviation over 10 replicate analyses and CI for 95% confidence interval computed following Cornuet et al. (2008).

By choosing among different competing scenarios, each analysis identifies the most probable source(s) for a given introduced population. The results come in the form of differing levels of support for the competing introduction models. It is thus worth stressing here that the identification of the most probable source population *X* (from among a finite set of possible source populations) does not necessarily mean that the introduced population *Y* originated from exactly location *X*, but rather that the most probable origin of the founders of the invasive population sampled at site *Y* is a population genetically similar to the source population sampled at site *X*. However, for sake of concision, we will use hereafter the simplified terminology that the most probable origin of *Y* is *X*.

After the early invasion of Hawaii (1980), the first recorded introduction was to the genetically heterogeneous western US, and thus that is where our main analyses start (analyses 1a–1d, table 2). We considered the three western US sample sites in separate analyses, and we found in each case that the best scenario included genetic admixture between Asia and Hawaii as sources (table 2; analysis 1a–1c, mean posterior probability of the best scenario *P *= 0.999). We subsequently tested if this common admixture pattern resulted from a single or multiple introduction events from Asia and/or Hawaii, including the possibility of local colonization events among western US sites (analysis 1d). The best invasion scenario for the western US included an initial admixture event with Asia and Hawaii as probable sources in northern California (site US-Wat; admixture event A1 in fig. 1), followed by (i) a local colonization southward into San Diego (US-SD) and northward into Oregon state (US-Sok), and (ii) a secondary introduction event from Hawaii to Oregon (US-Sok) (A2 event in fig. 1; *P = *0.690). This result is supported by various genetic clustering results (fig. 1, supplementary figs. S1 and S2, Supplementary Material online) as well as raw *F*-statistics values which indicate high differentiation level between US-Sok and other continental US sample sites and a substantially lower *F*_ST_ between US-Sok and Hawaii (supplementary table S5, Supplementary Material online). Once the invasion history was resolved for the western US group, we inferred the most probable source(s) of the invasive eastern US and European genetic groups, using two independent analyses (analyses 2a and 2b, table 1 and supplementary table S2, Supplementary Material online). We found evidence for a single origin for the eastern US group corresponding to an intra-continental spread from the San Diego area (*P *= 0.779, table 2).

**Table 3:** Bottleneck Severity in Invasive Populations of *D. suzukii*.

		Prior 1					Prior 2				
Introduction Type	Sample Site	Mean	Median	Mode	q5%	q95%	Mean	Median	Mode	q5%	q95%
Extra continental	US-Haw	0.517	0.500	0.495	0.326	0.755	0.490	0.484	0.477	0.382	0.615
	US-Wat	0.181	0.138	0.114	0.041	0.448	0.143	0.127	0.118	0.059	0.275
	IT-Tre	0.268	0.179	0.171	0.059	0.837	0.236	0.189	0.172	0.101	0.553
	BR-PA	0.158	0.126	0.104	0.020	0.407	0.208	0.193	0.172	0.067	0.399
	FR-Reu	0.259	0.215	0.186	0.051	0.626	0.245	0.214	0.190	0.069	0.534
Intra-continental	US-SD	0.135	0.117	0.106	0.039	0.283	0.117	0.104	0.091	0.053	0.224
	US-NC	0.089	0.067	0.061	0.015	0.230	0.111	0.098	0.090	0.046	0.218
Extra + intra continental	US-Sok	0.116	0.099	0.088	0.027	0.250	0.119	0.106	0.099	0.052	0.229
	GE-Dos	0.189	0.177	0.155	0.061	0.356	0.205	0.189	0.171	0.088	0.372
											
Prior values		0.628	0.199	NA	0.021	1.904	0.517	0.500	NA	0.326	0.754

Note.— Extra-continental introductions correspond to a long distance introduction from a source located apart from the continent of the focal population, intra-continental introduction corresponds to an introduction event from a source located on the same continent than the focal population, and Extra + Intra continental introduction corresponds to a combination of the two types of sources. Mean, median and mode estimates as well as bounds of 90% credibility intervals (q5% and q95%), are indicated for each bottleneck severity parameter. We roughly classified the estimated bottleneck severity values into three classes (represented here by the three shades of gray): weak (i.e., median value of bottleneck severity < 0.12, in light gray), moderate (0.12 < bottleneck severity < 0.22, in gray), and strong (i.e., bottleneck severity > 0.3 in dark gray). The set of sample sites used for the ABC estimations presented here include: (i) for the native area: Japan (JP-Tok + JP-Sap), South-East China (CN-Nin) and North-East China (CN-Lan + CN-Lia), and (ii) for the invaded range: US-Wat, US-Sok and US-SD for western US, US-NC for eastern US, IT-Tre for southern Europe, GE-Dos for northern Europe, BR-PA for South-America (Brazil) and FR-Reu for La Réunion island. Code names of the sample sites are the same as in fig. 1, and supplementary table S1, Supplementary Material online in which bottleneck severity classes are also given for each sample site. See supplementary table S6, Supplementary Material online for results on a different set of representative sample sites.

**Fig. 2:**
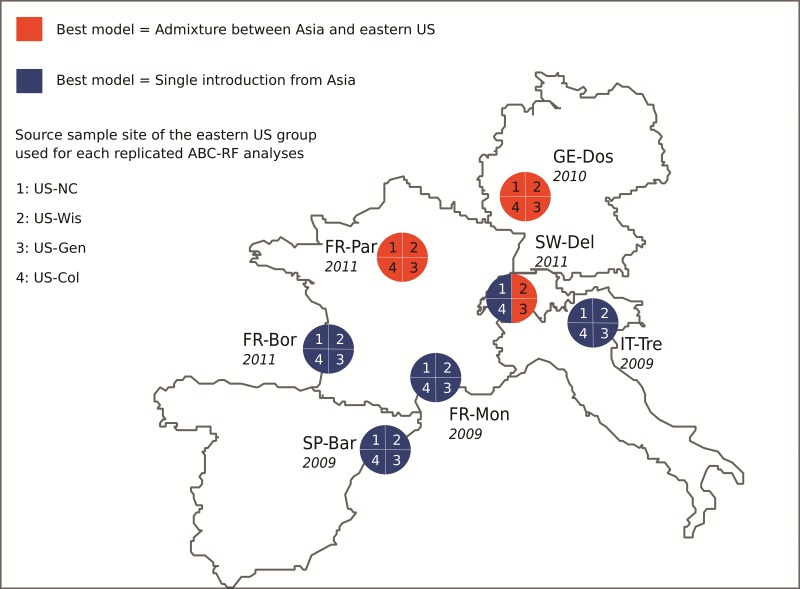
Admixed or non-admixed origin of *D. suzukii* in Europe inferred for each sample sites. Potential source populations include (among others) the Asian native range (represented by the Japanese sample site JP-Tok) and the invasive genetic group from eastern US represented by one of the four invasive sample sites collected in this area (fig. 1). Four replicate independent ABC-RF treatments corresponding to the analysis 3b (table 2) were hence carried out for each targeted European sample site using one of the four eastern US sample site. The treatments labeled 1, 2, 3, and 4 in the pies of the figure have been carried out with the sample sites US-NC, US-Wis, US-Gen, and US-Col, respectively. A pie quarter in blue indicates that the best scenario corresponds to a single introduction event from Asia. A pie quarter in red indicates that the best scenario corresponds to an admixture event between Asia and eastern US. Dates in italic correspond to the dates of first record of the European sample sites.

The most probable origin for Europe was an independent introduction from the Asian native range (*P *= 0.716, table 2). The European and North American invasions occurred nearly simultaneously, and thus we also explored gene flow between the two regions. Europe was never selected as a source for the eastern US (analysis 3a, *P *= 0.744), no matter which sets of sample sites were considered. In contrast, we found some evidence of asymmetrical gene flow from the eastern US into at least some European locations. When considering the northern European sample site from Germany (GE-Dos) as a target, we found that the best scenario included an admixed origin with Asia and the eastern US (supplementary table S4, Supplementary Material online, analysis 3b, *P* = 0.488 and *P* = 0.501 for prior set 1 and 2, respectively). In contrast, the best scenario did not include such admixture event when considering the southern European sample site from Italy (IT-Tre) as a target (table 2, analysis 3b, *P *= 0.510). We therefore replicated analysis 3b on all possible combinations of European and eastern US sample sites to assess the robustness of this result and evaluate the possibility of a geographic pattern for admixture events. For each of the European sample sites, figure 2 summarizes which replicate analyses selected a scenario including an admixture between eastern US and Asia or a scenario of a single introduction from Asia (and see supplementary fig. S3, Supplementary Material online, for results using an alternative representative sample site for the native range). Results tended to indicate an uneven spatial distribution of the presence of genes of eastern US origin in Europe, following roughly a north-south gradient. More specifically eastern US alleles were present in northern Europe and absent, or at least not detected by our model choice method, in southern Europe. We then further tested whether the observed admixture in the north corresponded to the mixing of eastern US genes with southern European genes originating from the initial introduction event from Asia versus the mixing of eastern US genes with Asian genes introduced through a separate secondary introduction event from Asia in northern Europe (analysis 4). The best scenario was clearly that of an admixture between eastern US genes and southern European genes, which themselves had a probable origin as part of the initial introduction event from Asia (*P *= 0.998; A3 event in fig. 1).

Finally, we found that the sampled population from Brazil originated from North America, with genetic admixture between *D. suzukii* individuals from the southwest and eastern regions of the US (analysis 5a; *P *= 0.631; A4 event in fig. 1). Regarding the most recent invasive population from La Réunion, we found that this island population originated from Europe, with admixture between individuals from northern and southern Europe (analysis 5b; *P* = 0.500; A5 event in fig. 1).

### Comparison of Model Choice Analyses Using ABC-RF versus ABC-LDA

For all 11 analyses, ABC-LDA performed well when using large reference tables (i.e., 500,000 simulations per scenario; table 2 and supplementary table S3, Supplementary Material online). Indeed, the prior error rates for ABC-LDA with large reference tables were smaller than the prior error rates for ABC-RF with small reference tables (paired *t*-test, *t*_10 _=_ _2.7, *P* = 0.02). However, ABC-RF performed better than ABC-LDA when using small reference tables (only 10,000 simulations per scenario), having lower prior error rates (paired *t*-test, *t*_10 _=_ _11.6, *P* < 0.0001, table 2 and supplementary table S3, Supplementary Material online). The difference in prior error rate was particularly marked for the complex analyses, i.e., those in which many introduction scenarios were compared and many summary statistics were computed. For instance, for the analyses 5a and 5b (21 compared scenarios and 424 summary statistics computed), prior error rates were 0.217 and 0.221 for ABC-RF versus 0.417 and 0.425 for ABC-LDA.

Regarding computational effort, we found that, for a given observed dataset, an ABC-RF treatment to select the best scenario and compute its posterior probability required a ca. 50 times shorter computational duration than when processing a ABC-LDA treatment with a traditional reference table of large size (i.e., 500,000 simulated datasets per scenario). Moreover, estimation of prior error rates with ABC-RF took only a few additional minutes of computation time for 10,000 pseudo-observed datasets whereas with ABC-LDA it lasted several hours to several days (depending on the analysis processed) for 500 pseudo-observed datasets.

The same invasion scenario had the highest probability for all 11 analyses carried out using ABC-RF and ABC-LDA with large number of simulated datasets (table 2 and supplementary table S3, Supplementary Material online). Posterior probabilities of the best scenario estimated using ABC-RF were not systematically higher (or lower) than those using ABC-LDA with large number of simulated datasets. In particular, there was no clear trend of overestimation of posterior probability when using ABC-LDA with large number of simulated datasets versus ABC-RF, a potential bias suggested by Pudlo et al. (2016). On the other hand, we found evidence of instability in the estimation of posterior probabilities and hence model selection when using ABC-LDA with a small numbers of simulated datasets in the reference table (i.e., 10,000 simulations per scenario as for ABC-RF). In this case, the scenario with the highest probability was different from that selected using either ABC-LDA with the large reference table or ABC-RF, in four and two of the 11 analyses when using the prior sets 1 and 2, respectively (results not shown).

### Refining the Origins of the Primary Introduction Events from the Native Area

Additional ABC-RF analyses were run to determine which geographical area in the native range was the most likely origin of each of the three primary introductions (i.e., in Hawaii, western US, and Europe). The most probable origin of the Hawaiian invasive population was Japan, with a mean posterior probability of 0.959 (origin O1 in fig. 1). For Watsonville (western US, sample site US-Wat), the most probable source of the primary introduction from Asia was southeast China (origin O2 in fig. 1; *P *= 0.860). Finally, for Europe, the most probable source of the primary introduction from Asia was northeast China (origin O3 in fig. 1; *P *= 0.855). Similar posterior probabilities were obtained using the prior set 1 (values presented above) and the prior set 2 (results not shown). For inferences regarding Europe, similar posterior probabilities were also obtained using various sets of sample sites from Europe and the eastern US (results not shown).

### Estimation of Parameter Distributions for Admixture Rate and Bottleneck Severity

We used the most probable worldwide invasion scenario (fig. 1) to estimate the posterior distributions of parameters related to bottleneck severity associated to the foundation of new populations and genetic admixture rates between differentiated sources (i.e., A1–A5 events in fig. 1).

The posterior distributions parameters relating to bottlenecks and admixture substantially differed from the priors, indicating that genetic data were informative for such parameters (tables 3 and 4; and see supplementary tables S6 and S7, Supplementary Material online for results using an alternative set of representative sample sites). Regarding bottleneck severity, we found that bottlenecks tended to be more severe for populations founded by individuals originating from a source located on a different continent (extra-continental origin) than for populations founded by individuals originating from a source located on the same continent (intra-continental origin; table 3 and fig. 1). The bottleneck severity for populations founded by individuals corresponding to a combination of the two types of sources (extra- and intra-continental origins) was either weak or moderate. The strongest bottleneck severity was found for the first invasive population in Hawaii, which showed the lowest level of genetic variation among all invasive populations. Bottleneck severity values were globally slightly stronger in European than in US populations, suggesting a smaller number of founding individuals and/or a slower demographic recovery in European populations.

**Table 4:** Posterior Distributions of Admixture Rates for the Five Admixture Events Inferred for the Final Worldwide Invasion Scenario Described in figure 1.

	Admixture Event	Admixture Rate (gene fraction from pop x)	Mean	Median	Mode	q5%	q95%
Prior 1	A1 (US-Wat = US-Haw + CN-Nin)	*r*_US-Wat_ (China – CN-Nin)	0.759	0.761	0.774	0.659	0.854
	A2 (US-Sok = US-Haw + US-Wat)	*r*_US-Sok_ (USA – US-Wat)	0.237	0.230	0.227	0.092	0.400
	A3 (DE-Dos = IT-Tre + US-NC)	*r*_DE-Dos_ (USA – US-NC)	0.286	0.278	0.266	0.112	0.481
	A4 (BR-PA = US-NC + US-SD)	*r*_BR-PA_ (USA – US-SD)	0.467	0.461	0.442	0.187	0.754
	A5 (FR-Reu = IT-Tre + GE-Dos)	*r*_FR-Reu_ (Europe – GE-Dos)	0.388	0.380	0.426	0.132	0.670
Prior 2	A1 (US-Wat = US-Haw + CN-Nin)	*r*_US-Wat_ (China – CN-Nin)	0.761	0.762	0.766	0.684	0.833
	A2 (US-Sok = US-Haw + US-Wat)	*r*_US-Sok_ (USA – US-Wat)	0.224	0.219	0.199	0.114	0.350
	A3 (DE-Dos = IT-Tre + US-NC)	*r*_DE-Dos_ (USA – US-NC)	0.312	0.306	0.284	0.152	0.487
	A4 (BR-PA = US-NC + US-SD)	*r*_BR-PA_ (USA – US-SD)	0.422	0.418	0.429	0.247	0.613
	A5 (FR-Reu = IT-Tre + GE-Dos)	*r*_FR-Reu_ (Europe – GE-Dos)	0.370	0.368	0.356	0.178	0.569

Note.— Admixture events are denoted as in figure 1. Each admixture rate parameter *r* points to the name of the admixed site and corresponds to the fraction of genes originating from the source site in parentheses (1 − *r* genes originate from the other source site). Mean, median, and mode estimates as well as bounds of 90% credibility intervals (q5% and q95%) are indicated for each admixture parameter. Estimations assuming the prior set 1 and the prior set 2 (supplementary table S8, Supplementary Material online) are provided. The set of representative sample sites used for the ABC estimations presented here is the same than in table 3. Code names of the population sites are the same as in fig. 1 and supplementary table S1, Supplementary Material online. See supplementary table S7, Supplementary Material online for results on a different set of representative sample sites.

Regarding admixture (table 4), we found that: (i) for the first recorded invasive population in western US (i.e., Watsonville; sample site US-Wat), the genetic contribution from China was substantially larger than that from Hawaii (median value of 0.759), (ii) for the western US population US-Sok, the secondary genetic contribution from Hawaii was small compared to that from Watsonville (median value of 0.237), and (iii) the sample sites from northern Europe (e.g., GE-Dos in Germany) contained a rather low proportion of genes originating from the eastern US (median value of 0.286). We found more balanced admixture rates for the populations from Brazil (BR-PA) and La Réunion (FR-Reu), but information on this parameter was less accurate in these cases as indicated by large 90% credibility intervals.

### Model-Posterior Checking

Like any model-based methods, ABC inferences do not reveal the “true” evolutionary history, but allows choosing the best among a necessarily limited set of scenarios that have been compared and to estimate posterior distributions of parameters under this scenario. How well the inferred scenario-posterior combination matches with the observed dataset remains to be evaluated using an ABC model-posterior checking analysis. When applying such an analysis on the final invasion scenario detailed in figure 1, we found that, when considering the prior set 1, only 54 of the 1141 summary statistics used as test quantities had low posterior predictive *P*-values (i.e., 0.002 < *ppp*-values < 5%). Moreover, none of those *ppp*-values values remained significant when correcting for multiple comparisons (Benjamini and Hochberg 1995). Similar results were obtained when assuming the prior set 2 and when considering different sets of representative sample sites in our analyses (results not shown). These findings show that the final worldwide invasion scenario (fig. 1, with associated parameter posterior distributions) matches the observed dataset well. In agreement with this, the projections of the simulated datasets on the principal component axes from the final model-posterior combination were well grouped and centered on the target point corresponding to the observed dataset (supplementary fig. S4, Supplementary Material online). Due to the modest size of the dataset (i.e., 25 microsatellite loci), however, only situations of major inadequacy of the model-posterior combination to the observed dataset are likely to be identified.

## Discussion

In the present paper, we decipher the routes taken by *D. suzukii* in its invasion worldwide, evaluating evidence of bottlenecks and genetic admixture associated with different introductions, and we quantify the efficiency of ABC-RF relative to the more standard ABC-LDA method for choosing among introduction scenarios.

### A Complex Worldwide Invasion History

Prior to this study and that of Adrion et al. (2014), our understanding of the worldwide introduction pathways of *D. suzukii* was based on historical and observational data, which were incomplete and potentially misleading. Our results indicate three distinct introductions from the native range—to Hawaii, the western side of North America and western Europe, which accords well to findings of Adrion et al. (2014) from a different set of markers (X-linked sequence data). Both the western North American introductions and at least some European populations show signs of admixture. Hawaii and China both appear to have contributed to the western North American introductions, which was in turn the most probable source for the introduction into eastern North America. Similarly, China and to a lesser extent eastern North America both appear to have contributed to the European introduction, but mostly in northern European areas with respect to the eastern North American contribution. The almost simultaneous invasion of North America and Europe from Asia could be due to increased trade between these areas facilitating transport of this species between regions. Alternatively, or in addition, adaptation to human-altered habitats (in this case, changes in agricultural practices) within the native range of the species, could have promoted invasion to similarly altered habitats worldwide (i.e., anthropogenicaly induced adaptation to invade; Hufbauer et al. 2012). However, the two invaded continents were most likely colonized by flies from distinct Chinese geographic areas (fig. 1), requiring that adaptation to agriculture occurred concomitantly at several locations within the native range. This is possible, but not evolutionarily parsimonious, and further data would be required to test this explanation. Subsequent introductions from the three primary founder locations to other locations result in a complex history of invasion. Both primary and secondary introductions show signs of demographic bottlenecks, and several invasive populations have multiples sources and thus experienced genetic admixture between differentiated native or invasive populations.

The genetic relationships between worldwide *D. suzukii* populations define the genetic variation they harbor, and may continue to shape further spread of alleles among populations. Gene flow among populations through continuous dispersal or more punctual admixture events permits the dissemination of allelic variants and new mutations, with dramatic consequences for evolutionary trajectories (Lenormand 2002). We found evidence for asymmetric genetic admixture from North American towards (northern) European populations. If this signal of admixture reflects recurrent and on-going dispersal events rather than a single past secondary introduction event, then variants that arise in North America would be more likely to spread to Europe than the reverse. Discriminating between ongoing and past dispersal events is tricky, however, especially for recent invasions (Benazzo et al. 2015). If the occurrence of recurrent gene flow is confirmed, then *D. suzukii* populations from Europe may not lag behind American ones in evolutionary terms. From an applied perspective, if resistance to control practices emerges in North America, special efforts to stop the importation of *D. suzukii* to Europe would help prevent the evolution of resistance in Europe, and thus reduce damage to crops (Caprio and Tabashnik 1992).

### Advances in Methods for Discriminating among Complex Models

To the best of our knowledge, the present study is the first to use the recently developed ABC-RF method (Pudlo et al. 2016) to test competing models characterized by different levels of complexity. To compare ABC-RF to a more standard ABC method, we carried out a subset of model choice analyses using both ABC-RF and ABC-LDA (Estoup et al. 2012). We ran ABC-LDA analyses in two ways: with standard sized large reference tables (500,000 simulated datasets per scenario) and with small reference tables (10,000 simulated datasets per scenario, the same size as used for ABC-RF). The performance of ABC-LDA (in terms of prior error rate) in choosing among introduction scenarios was slightly better than for ABC-RF, when using large reference tables. Furthermore, for ABC-LDA with large reference tables confidence in model choice (in terms of posterior probability) was relatively comparable to ABC-RF with small reference tables. Thus, when analyses are relatively simple or computational resources are not limiting, ABC-LDA can provide as robust inferences of invasion scenarios as ABC-RF.

In contrast, we found that ABC-RF out-performed ABC-LDA when using similarly small reference tables for both types of analyses (10,000 simulated datasets per scenario). ABC-LDA also was unstable, choosing different scenarios in different replicate analyses, when using small reference tables. These empirical findings correspond well with those of Pudlo et al. (2016), using simple models for which the true posterior probabilities could be calculated. Pudlo et al. (2016) demonstrated that ABC-RF provided more reliable posterior probability of the best (true) scenario than standard ABC methods when using the same (small) number of simulated datasets in the reference table (see supplementary fig. S3 in Pudlo et al. 2016). In our work, the difference in performance between the two methods was most pronounced for complex analyses (e.g., when comparing more than 10 scenarios using more than 100 summary statistics). Thus, when analyses are not simple, or computational resources are finite, ABC-RF provides more robust inferences of invasion scenarios than ABC-LDA.

The lower computation cost of ABC-RF was also evident. For a given observed dataset, selecting the best supported scenario and computing its posterior probability was ca. 50 times faster than when processing an ABC-LDA treatment with a traditional reference table of large size. Moreover, estimation of prior error rates with ABC-RF took only a few additional minutes of computation time whereas it lasted several hours to several days with ABC-LDA. The consequence of low computational costs is not simply in computer time. Rather, the efficiency of ABC-RF analyses allowed us to run replicate analyses on various sample sets, even for the most complex *D. suzukii* invasion scenarios. This replication is critical in evaluating the robustness of our statistical inferences.

We found that, at least for some ABC-RF analyses, the posterior probability of the best scenario and the global statistical power to choose among alternative invasion scenarios (as measured by the prior error rate), were relatively low. For instance, in three of the 11 analyses (analyses 3b, 5a, and 5b) the best scenarios had relatively low probabilities (i.e., ranging from 0.500 to 0.630) and relatively high prior error rates (i.e., ranging from 0.300 to 0.400). Although replicate analyses carried out on different sample sets and prior sets pointed to the same best scenario for these three analyses, such low probability values and high prior error rates suggest a moderate level of confidence in the choice of model in these cases. Similarly, we found that the scenario choice leading to the conclusion of the presence versus absence of admixed genes from the eastern US in various European sample sites relied on probability values that were sometimes as low as 0.500. Moreover, in a minority of cases, the best scenario was different depending on the sample site considered as representative of the eastern US or the native area. The observed pattern of an uneven spatial distribution of the presence of genes of eastern US origin in Europe, following roughly a north to south gradient, should hence be taken cautiously. The analyses of additional European sample sites are needed to clarify this issue. The above inferential uncertainties also most likely find their sources in the relatively small number of markers genotyped relative to the number, complexity, and similarity of some of the competing models. Such results indicate that there is room for improving the robustness of our inferences at least for a subset of our analyses.

### Bottlenecks and Genetic Admixture

We found strong evidence that bottlenecks and admixture both occurred during the course of the invasion of *D. suzukii*. A first sign of bottlenecks is a reduction in neutral genetic diversity. We found that all invasive *D. suzukii* populations were characterized by significantly lower genetic variation than native ones, with a loss of 6.4% (US-Col) to 23.3% (US-Haw) of heterozygosity and of 27.3% (US-Wat) to 54.2% (US-Haw) of allelic diversity, respectively (supplementary fig. S5, Supplementary Material online). Adrion *et al.* (2014) find a similar significant loss of diversity in their dataset focused on gene sequences. Dlugosch and Parker (2008) and Uller and Leimu (2011) reviewed studies of neutral genetic diversity in a large number of species of animals, plants, and fungi and compared nuclear molecular diversity within introduced and source populations. Overall, they found that a loss of variation was the most frequent feature in invasive populations. However, reductions in genetic variation were on average modest (e.g., average loss of 18.7% and 15.5% of heterozygosity and allelic diversity, respectively; Dlugosch and Parker 2008). The reductions observed in our study in invasive *D. suzukii* populations were thus in the same range for heterozygosity but higher for allelic diversity (cf. average loss of 11.1% and 34.5% of heterozygosity and allelic diversity, respectively). A larger loss of allelic diversity than heterozygosity after a bottleneck is nevertheless expected by theory (Nei et al. 1975).

The type of introduction pathway explains, at least partly, the severity of the reduction in genetic diversity. Bottlenecks tended to be less severe for populations founded by individuals from the same continent than for populations founded by individuals originating from a different continent. A simple explanation for such pattern is that, due to greater proximity, populations originating from the same continent are likely to be initially founded by a larger numbers of individuals and also to experience recurrent gene flow. Populations founded by individuals from both the same and different continents experienced weak or moderate reductions in diversity, suggesting that multiple introduction pathways restore diversity.

In agreement with previous studies on invasions on similarly large geographical scales (e.g., Lombaert et al. 2014 and reference therein), we found that admixture events were frequent in the worldwide invasion history of *D. suzukii*. We identified at least five admixture events for which sources were native and/or invasive populations (i.e., A1–A5 events in fig. 1). The genetic contributions of the sources were unbalanced, except for the populations from Brazil and La Réunion; but genetic information regarding admixture was considerably less accurate in the latter cases, which involved weakly differentiated source populations.

We have provided here the information necessary to evaluate whether bottlenecks and admixture have influenced outcomes in this invasion. Quantitative genetics studies in the lab focusing on the key source, bottlenecked, and admixed populations could now examine the fitness and adaptive potential of those groups (Lavergne and Molofsky 2007; Facon et al. 2008; Turgeon et al. 2011).

## Conclusions and Perspectives

Understanding invasion pathways provides key information for understanding the role of evolutionary processes in biological invasions. For example, only does our research reveal sources of invasive populations for further relevant comparisons using quantitative genetics studies in the lab. Additionally, we found that the invasion of Europe by *D. suzukii* was distinct from that of North America, with limited and asymmetrical gene flow between these two main invaded areas. This situation provides the opportunity to evaluate evolutionary trajectories in replicate into two separate temperate climate regions, similarly to research by Gilchrist et al*.* (2001, 2004) on the pace of clinal evolution in the invasive fruit fly *Drosophila subobscura*. There are also distinct invasion pathways for areas with warmer climates (i.e., Hawaii, La Réunion, and Brazil) that will make interesting comparisons.

Retracing invasion routes and making inferences about demographic processes is only possible if there is adequate polymorphism within populations and significant genetic differentiation among them. As is evident here, microsatellite data can provide enough variation to reveal important pathways. We concur with Adrion et al. (2014), however, that genome-wide data (i.e., next generation sequencing approaches) will be tremendously powerful in further discriminating among complex invasion scenarios (see also Cristescu 2015, Estoup et al. 2016). Because of the reduced computational resources demanded by ABC-RF, this method will be particularly useful for analysis of massive single nucleotide polymorphism datasets (Pudlo et al. 2016). In addition to the selectively neutral demographic inference leading to the reconstruction of routes of invasion, population (and quantitative) next generation sequencing approaches are quite promising for studying the evolution of phenotypic traits in natural populations (Wray 2013; Gautier 2015). Specifically, they can be used to better understand the genetic architecture of traits underlying invasion success (Bock et al. 2015). *Drosophila suzukii* is a good species for such research given (i) its short generation time and viability in laboratory conditions, which facilitate experimental approaches to study quantitative traits of interest (Asplen et al. 2015) and (ii) the availability of annotated genome assemblies for this species (Chiu et al. 2013; Ometto et al. 2013), along with the huge amount of genomic resources available in its close relative species *D. melanogaster* (Groen and Whiteman 2016).

## Materials and Methods

### Sampling and Genotyping

Adult *D. suzukii* were sampled from the field at a total of 23 localities (hereafter termed sample sites) distributed throughout most of the native and invasive range of the species (supplementary table S1, Supplementary Material online and fig. 1). Samples were collected between 2013 and 2015 using baited traps and sweep nets, and stored in ethanol. Native Asian samples consisted of a total of six sample sites including four Chinese and two Japanese localities. Samples from the invasive range were collected in Hawaii (1 sample site), Continental US (7 sites), Europe (7 sites), Brazil (1 site), and in the French island of La Réunion (1 site). For each sample site, 15–44 adult flies were genotyped at 25 of the 28 microsatellite loci described in Fraimout et al. (2015), resulting in a total of 685 genotyped individuals. Three microsatellite loci from Fraimout et al. (2015) (i.e., loci DS31, DS42 and DS45) were not included due to the presence of null alleles at frequencies > 10% in some sample sites (results not shown). DNA extraction and PCR amplification as well as allele scoring were performed following the methodology detailed in Fraimout et al. (2015).

### General Framework for Defining Focal Population Groups and Sets of Scenarios to Be Compared Using ABC

One of the many challenges of ABC analyses is to optimally define sets of sample sites to be processed chronologically as potential sources and targets in a way that do not hamper the computational effort. As stated in Estoup and Guillemaud (2010) and Lombaert et al. (2014) the number and complexity of competing scenarios is proportional to the number of genetic groups to be accounted in ABC analyses. Following Lombaert et al. (2014), we used the results obtained from three genetic clustering methods along with historical and geographical information to define genetic groups that could include several sample sites (supplementary appendix S1, Supplementary Material online). We defined seven main genetic groups from our set of 23 sample sites: Asia, Hawaii, western US, eastern US, Europe, Brazil, and La Réunion. To make the computations less intensive with respect to computer resources, ABC treatments were carried out using one representative sample site for each genetic group per replicate analysis. In other words, when a genetic group included more than two sample sites, we considered the two most differentiated sample sites (i.e., with the highest *F*_ST_ values in supplementary table S3, Supplementary Material online) as representative of the group: CN-Shi and JP-Tok for the group Asia, all three sample sites for the genetically heterogeneous Western US group, US-NC and US-Wis for the group eastern US, GE-Dos, and IT-Tre for the group Europe. Each of the sample sites retained (i.e., the representative sample sites) was included alternatively in the sample set considered for ABC treatments. This makes it possible to replicate analyses of scenario choice on different sample sets representing most of the genetic variation within and between genetic groups. All ABC analyses described in table 1 were thus replicated with all possible combinations of representative sample sites available for each genetic group. This allowed us to test the robustness of scenario choices with respect to the sample sets considered as representative of genetic groups.

### Nested ABC Analyses Processed Sequentially

Following Lombaert et al. (2014), we used historical information (i.e., dates of first observation of each invasive populations; supplementary table S1, Supplementary Material online) to define 11 sets of competing introduction scenarios that were analyzed sequentially. The first set of compared scenarios considers the second oldest invasive population as target (the oldest one necessarily originating from the native range) and determines its introduction history. Step by step, subsequent analyses use the results obtained from the previous analyses, until the most recent invasive populations are considered. Each of the 11 analyses included a set of compared introduction scenarios where the target invasive population (i.e., the focal population) can either directly derive by a single split (i.e., introduction event) from one of the historically compatible source populations, or be the result of an admixture event between one of all possible combinations of pairwise sources. For instance, in a model with one target and two possible sources, three scenarios could be formalized with the target population being derived from a single source population, from the other possible source, or from an admixture between the two sources (see supplementary fig. S6 for an illustration and supplementary table S2, Supplementary Material online for details).

The first set of scenario choice analyses 1a–1d aimed at making inferences about the introduction pathways for the three sample sites in western US (table 1). We first evaluated the Asian or Hawaiian or admixed Asian + Hawaiian origin of each three western US sample sites. We then refined our modeling by formalizing seven competing scenarios describing the possible relationships among the three western US sample sites and their extra-continental sources (i.e., Asia and Hawaii; analysis 1d). Once the invasion history was resolved for the western US group, we sought to identify the population source(s) of the eastern US and European genetic groups, independently. Dates of first records in these two groups widely overlap and the possibility that the western US was a common source for both areas could not be ruled out. We therefore compared in analysis 2a a set of six scenarios to assess whether the eastern US group directly derived from Asia, Hawaii, or western US or if it resulted from an admixture event between one pair of such sources. A similar set of six scenarios was used to assess the most likely origin of European populations, independently from the eastern US group (analysis 2b).

Due to the results of analyses 2a and 2b and due to the overlap in first record dates for eastern US and Europe, we then performed two analyses to investigate the possibility of gene flows (i.e., admixture) between the eastern US and European groups, considering eastern US as target population with Europe as potential source and *vice versa* (analyses 3a and 3b, reciprocally). Analysis 4 aimed at clarifying the admixture in northern Europe sample sites identified in analysis 3b. We tested whether this admixture occurred between eastern US and a secondary introduction from Asia, or between individuals from eastern US and southern Europe. Finally, we investigated the origins of the most recent invasive populations in Brazil and La Réunion, separately, with all older groups and pairwise admixtures as potential sources (analyses 5a and 5b). Note that in agreement with the genetic clustering results (fig. 1, supplementary appendix S1 and figs. S1 and S2, Supplementary Material online) we did not test for the possibility of Brazil being the source of La Réunion, and reciprocally.

### Historical, Demographic, and Mutational Parameters

For all scenarios formalized for ABC analyses, we modeled an introduction event as a divergence without subsequent gene-flow from the source population (or two source populations in case of admixture) at a time corresponding to the date of first record in the invaded area translated into a number of generations (assuming 12 generation per year; Lin et al. 2014). The divergence event was immediately followed by a bottleneck period characterized by a lower effective population size, and a straight return to a stable (large) effective population size. We modeled our uncertainty regarding the identity of the source populations in the slightly structured native Asian area by including native “ghost populations” (i.e., unsampled populations) in our scenarios. Such unsampled native populations were modeled as a single split from an ancestral Asian population (without any change in effective population size) at a time defined by a loose flat prior, which includes zero at the lower bound (supplementary table S8, Supplementary Material online). See Lombaert et al. (2014) and references therein for justifications of using unsampled populations when modeling invasion scenarios. A detailed illustration of the above modeling design is provided in supplementary fig. S6, Supplementary Material online.

Prior distributions for historical, demographical, and mutational parameters were defined taking into account the historical and demographic parameter values available from empirical studies on *D. suzukii* (Hauser 2011; Lin et al. 2014; Asplen et al. 2015) and microsatellite mutational data on *D. melanogaster* (Schug et al. 1998). We considered a first set of prior distributions (hereafter termed prior set 1), which was composed of rectangular (i.e., bounded uniforms) distributions (see supplementary table S8, Supplementary Material online). To evaluate the robustness of our ABC inferences to prior choice, we also considered a second set of more peaked prior distributions (hereafter termed prior set 2; supplementary table S8, Supplementary Material online).

### Model Choice Using ABC-RF and ABC-LDA

For all ABC-RF and ABC-LDA analyses, we used the software DIYABC v.2.1.0 (Cornuet et al. 2014) to simulate datasets constituting the reference tables. A reference table includes a given number of datasets that have been simulated for different scenarios using parameter values drawn from prior distributions, each dataset being summarized with a pool of statistics.

Following Pudlo et al. (2016), ABC-RF treatments were processed on reference tables including 10,000 simulated datasets per scenario. Datasets were summarized using the whole set of summary statistics proposed by DIYABC (Cornuet et al. 2014) for microsatellite markers, describing genetic variation per population (e.g., number of alleles), per pair (e.g., genetic distance), or per triplet (e.g., admixture rate) of populations, averaged over the 25 loci (see the supplementary table S9, Supplementary Material online for details about such statistics), plus the linear discriminant analysis (LDA) axes as additional summary statistics. The total number of summary statistics ranged from 39 to 424 depending on the analysis (table 2). When the number of scenarios in an analysis exceeded ten, we processed ABC-RF treatments on reference tables including 100,000 simulated datasets to avoid computer memory issues associated to the sub-bootstrapping procedure processed for reference tables with more than 100,000 simulated datasets; see the section *Practical recommendations regarding the implementation of the algorithms* in Pudlo et al. (2016). We checked that this number was sufficient by evaluating the stability of prior error rates (i.e., the probability to choose a wrong model when drawing model index and parameter values into priors) and posterior probabilities estimations on 80,000, 90,000, and 100,000 simulated datasets. The number of trees in the constructed random forests was fixed to *n* = 500; see Pudlo et al. 2016, Breiman 2001 for justifications of considering a forest of 500 trees and supplementary fig. S7, Supplementary Material online for an illustration. For each ABC-RF analysis, we predicted the best scenario, estimated its posterior probabilities and prior error rates over 10 replicate runs of the same reference table. We used the abcrf R package (v1.1.0; Pudlo et al. 2016) to perform all ABC-RF analyses. In supplementary appendix S2, Supplementary Material online, we provide several scripts written in R programming language (R Development Core Team 2008) for computing random forest analyses in R with the abcrf package, when starting from simulated datasets generated with DIYABC v.2.1.0.

For sake of methodological comparisons, we also duplicated a subset of analyses using a more standard ABC method (Beaumont et al. 2002; Cornuet et al. 2008) improved using the regression algorithms from Estoup et al. (2012) based on linear discriminant analysis (LDA) to avoid the curse of dimensionality and correlation among explanatory variables (i.e., multi-co-linearity) during the regression step. This method is called ABC-LDA. Following previous analyses of this type (Lombaert et al. 2014), ABC-LDA treatments were processed on reference tables including 500,000 simulated datasets per scenario. As for ABC-RF, datasets were summarized using the whole set of summary statistics proposed by DIYABC (Cornuet et al. 2014). Posterior probabilities associated with each scenario were estimated by polychotomous logistic regression (Cornuet et al*.* 2008), modified following Estoup et al. (2012), on the 1% of the simulated datasets closest to the observed dataset. We also estimated for each analysis a prior error rate over 500 simulated datasets drawn into priors using reference tables including 500,000 simulated datasets per scenario. To provide a fair comparison of classification error with respect to the computational effort, prior error rates were also estimated using reference tables including the same number of simulated datasets per scenario as for ABC-RF treatments (10,000 per scenario).

### Refining the Origins of the Primary Introduction Events from the Native Area

We refined our worldwide invasion scenario by assessing the most probable origin of the primary introductions from the Asian native range. To this aim, we carried out additional ABC-RF treatments to choose which geographical area in the native range was the most likely origin of each of the three primary introductions from Asia identified in the final invasion scenario, i.e., in Hawaii, western US (sample site US-Wat), and Europe (fig. 1). Capitalizing on the observed pattern of genetic differentiation among all Asian sample sites, we considered four possible geographical origins: O1 = Japan (represented by the sample sites JP-Sap + Jp-Tok that were pooled as they did not show any significant differentiation), O2 = South-East China (represented by the sample site CN-Nin), O3 = North-West China (represented by the sample sites CN-Lan + CN-Lia that were pooled as they did not show any significant differentiation), and O4 = South-West China (represented by the sample site CN-Shi). In each of these three model choice analyses (cf. one analysis per primary introduction), we considered the previously inferred introduction histories and compared four scenarios corresponding to variation of those histories that only differed by the geographical origin of the primary Asian introduction (models O1, O2, O3, and O4). We used the ABC-RF method with 10,000 datasets per model simulated using the prior set 1 or 2, and with three replications of the RF process. For inferences regarding Europe, we carried out ABC-RF treatments on various sets of sample sites from Europe and eastern US to further evaluate the robustness of our results.

### Parameter Estimation

For ABC parameter estimation, we considered a set of 12 key sample sites representative of the inferred final invasion history (fig. 1). This set of sample sites included the three native origins of primary introductions from the native range (O1, O2, and O3), the Hawaiian sample (US-Haw), the three western US samples (US-Wat, US-Sok, and US-SD), the eastern US sample US-NC, two European samples (IT-Tre and GE-Dos), the Brazilian sample (BR-PA), and the sample from La Réunion (FR-Reu). To evaluate the robustness of our parameter estimations with respect to sample sites we carried out a second analysis replacing the sample sites US-NC, IT-Tre, and GE-Dos by US-Wis, SP-Bar, and FR-Par, respectively. We also replicated parameter estimations using the prior set 1 and the prior set 2.

ABC-RF was developed to tackle the inferential issue of model choice. So far, no RF-based solution exists to estimate parameter distributions under a given model (Pudlo et al. 2016). Standard ABC algorithms for parameter estimation may suffer from the curse of dimensionality and correlation among explanatory variables (i.e., multi-co-linearity) during the regression step, and hence yield poor results when the number of statistics is much too large [reviewed in Blum et al. (2013)]. To avoid such potential problems as well as for the sake of simplicity and computation efficiency (i.e., statistical techniques for choosing summary statistics have not been implemented in DIYABC; Blum et al. 2013), we used a standard ABC methodological framework (Beaumont et al. 2002; Cornuet et al. 2008) applied to a subset of “expert-chosen” statistics proposed in DIYABC (Brouat et al. 2014; Lombaert et al. 2014) to estimate posterior parameter distributions under the final invasion scenario. We hence considered 95 summary statistics in total: the mean number of alleles and the mean genetic diversity (per locus and population sample), all pairwise *F*_ST_’s, and some crude estimates of admixture rates based on the five population triplets for which admixture events have been inferred (i.e., A1–A5 events described in fig. 1 corresponding to five AML statistics; Choisy et al. 2004, see the DIYABC 2.1.0 user-manual p16 for details about such statistics). Using parameter values drawn from the prior sets 1 or 2, we produced a reference table containing 10 million simulated datasets. Following Beaumont et al. (2002), we then used a local linear regression to estimate the parameter posterior distributions. We took the 10,000 (1^°^/_00_) simulated datasets closest to our observed dataset for the regression, after applying a logit transformation to parameter values. We found that considering instead the 1% closest simulated datasets and/or a larger sets of summary statistics had only weak effects on the estimated parameter distributions, at least for the subset of parameters we were interested in (i.e., admixture rates and bottleneck severity) (results not shown).

We focused our investigations on the parameters related to two types of demographic events that are considered to be potentially important drivers of invasion success: severity of the bottleneck associated with the foundation of new populations and genetic admixture (Estoup et al. 2016). Bottleneck severity was defined as the ratio between the duration of the bottleneck and the number of effective individuals during this period (Pascual et al. 2007). Following the final invasion scenario detailed in figure 1, we defined three categories of introduction situations: bottleneck severity for populations founded only by individuals originating from a different continent (e.g., sample sites US-Haw, US-Wat, IT-Tre, BR-PA, and FR-Reu), bottleneck severity for populations founded only by individuals originating from the same continent (e.g., sample sites US-SD and US-NC) and bottleneck severity for populations founded by a combination of the two types of sources (e.g., sample sites US-Sok and GE-Dos). Genetic admixture is evident when a population is inferred to have more than one distinct source (A1–A5 events in fig. 1).

### Model-Posterior Checking

We used the ABC model-posterior checking method implemented in DIYABC (Cornuet et al. 2010) to evaluate how well the final worldwide invasion scenario with associated parameter posterior distributions matches with the observed dataset. This method was largely inspired by statistical frameworks detailed in Gelman et al. (2003) and Cook et al. (2006). The principle is as follows: if a scenario-posterior combination fits the observed data correctly, then data simulated under this scenario with parameters drawn from associated posterior distributions should be close to the observed data. The lack of fit of the model to the data can be measured by determining the frequency at which test quantities measured on the observed dataset are extreme with respect to the distributions of the same test quantities computed from the simulated datasets (i.e., the posterior predictive distributions). In practice, the test quantities are chosen among the large set of ABC summary statistics proposed in the program DIYABC. For each test quantities (*q*), a lack of fit of the observed data with respect to the posterior predictive distribution can be measured by the cumulative distribution function values defined as Prob(*q*_simulated _<_ _*q*_observed_). Tail-area probability can be easily computed for each test quantityas Prob(*q*_simulated _<_ _*q*_observed_) and 1.0 − Prob (*q*_simulated _<_ _*q*_observed_) for Prob (*q*_simulated _<_ _*q*_observed_) ≤ 0.5 and > 0.5, respectively. Such tail-area probabilities, also named posterior predictive *P*-values (i.e., *ppp*-values; Meng 1994), represent the probabilities that the replicated data (simulated ABC summary statistics) could be more extreme than the observed data (observed ABC summary statistics). In practice, *ppp*-values can be interpreted as a guideline for tracking model-posterior misfit: a few *ppp*-values around 0.05 are not necessarily a problem whereas the presence of *ppp*-values around 0.001 is rather suspect. Hence, too many observed summary statistics falling in the tails of distributions, especially if some of those *ppp*-values are small (<0.001), cast serious doubts on the adequacy of the model-posterior combination to the observed dataset. Finally, because *ppp*-values are computed for a number of (often non-independent) test statistics, a method such as that of Benjamini and Hochberg (1995) can be used to control the false discovery rate (Cornuet et al. 2010).

We carried out the ABC model-posterior checking analysis on our observed microsatellite dataset as follows. From 10^7^ datasets simulated under the final invasion scenario detailed in figure 1 and using the same subset of 95 statistics previously retained for parameter estimation, we obtained a posterior sample of 10^4^ values from the posterior distributions of parameters, as described in the previous section *Parameter estimation*. We then simulated 10^4^ datasets with parameter values drawn with replacement from this posterior sample. As underlined in many text books in statistics (Gelman et al. 2003), it is advised against performing model checking using information that have already been used for training (i.e., model fitting; see also Cornuet et al. 2010 for illustrations on simulated datasets). Optimally, model-posterior checking should be based on test quantities that do not correspond to the summary statistics which have been used for obtaining the parameters posterior distributions. This is naturally possible with DIYABC as the package propose a large choice of summary statistics. Our set of test statistics therefore included the 1141 remaining single, pairwise and three-population summary statistics available in DIYABC that were not used for previous ABC parameter estimation.

## Supplementary Material

Supplementary data are available at *Molecular Biology and Evolution* online.

## Supplementary Material

Supplementary Data
