# Supplementary material for: Deciphering the Routes of invasion of Drosophila suzukii by Means of ABC Random Forest
Source: Mol Biol Evol. 2017 Feb 25;34(4):980–96. doi: 10.1093/molbev/msx050 (PMC5400373; doi:10.1093/molbev/msx050)
Supplement: Supplementary Data [file msx050_Supp.pdf]

Supplementary material for the manuscript: "Deciphering the routes of invasion of *Drosophila suzukii* by means of ABC random forest"

Antoine Fraimout[1], Vincent Debat[1], Simon Fellous[2], Ruth A. Hufbauer[2,3], Julien Foucaud[2], Pierre Pudlo[4], Jean-Michel Marin[5], Donald K. Price[6], Julien Cattel[7], Xiao Chen[8], Marindia Depra[9], Pierre François Duyck[10], Christelle Guedot[11], Marc Kenis[13], Masahito T. Kimura[14], Gregory Loeb[12], Anne Loiseau[2], Isabel Martinez-Sañudo [15], Marta Pascual[16], Maxi Polihronakis Richmond[17], Peter Shearer[18], Nadia Singh[19], Koichiro Tamura[20], Anne Xuéreb[2], Jinping Zhang[21] & Arnaud Estoup[2].

Corresponding author: Arnaud Estoup, UMR Centre de Biologie pour la Gestion des Populations (INRA ⁄ IRD⁄Cirad ⁄ Montpellier SupAgro), 755 avenue du Campus Agropolis, CS 30 016, 34988 Montferrier-sur-Lez cedex, France; email: arnaud.estoup@inra.fr

Short title: Reconstructing invasion routes using ABC random forest

Key words: *Drosophila suzukii*, invasion routes, random forest, approximate Bayesian computation, population genetics.

Table of contents

Appendix S1: Characterization of genetic diversity and structure within and between all genotyped sample sites and definition of genetic groups and representative sample sites for ABC analyses................3

Figure S1. Results from analysis using Discriminant Analysis of Principal Components (DAPC). 6

Figure S2. Genetic groups identified by STRUCTURE. 7

Figure S3: Admixed or non-admixed origin of D. suzukii in Europe inferred for each sample sites, using an alternative representative sample site for the native range..10

Figure S4. Principal component analysis of 1141 test quantities when processing ABC model-posterior checking for the final worldwide invasion scenario detailed in fig. 1..11

Figure S5. Genetic diversity in the invasive and native sample sites of D. suzukii. 12

Figure S6. Graphic representation of three ABC scenarios to infer the origin of the US-Wat sample (analysis 1a in table S1 and 2). 13

Figure S7. Evolution of the ABC-RF prior error rate with respect to the number of trees in the forest. 14

Table S1. Locations from which D. suzukii individuals were collected for these analyses, including the date of first observation at the site, and the year sampled..16

Table S2. Focal genetic group and origin of its source population(s) for each competing scenarios considered for each eleven ABC analyses..17

Table S3. Results of model choice analyses using ABC-RF and ABC-LDA, using the prior set 2................19

Table S4. Results of the ABC-RF analyses defined in table 2 for an alternative set of representative sample sites using prior sets 1 and 2. 20

Table S5. FST values between all pair of sample sites. 21

Table S6. Bottleneck severity in invasive populations of D. suzukii for an alternative set of representative sample sites. 22

Table S7. Admixture rates estimated for an alternative set of representative sample sites. 23

Table S8. Prior sets 1 and 2: historical, demographic and mutation parameters used for ABC analyses, with their interpretation and their prior distributions. 24

Table S9. Summary statistics used for model choice using ABC-RF and ABC-LDA...................................26

Appendix S2: R scripts to run ABC-RF model choice analyses using datasets simulated with the package DIYABC v2.1.0..28

Appendix S1: Characterization of genetic diversity and structure within and between all genotyped sample sites and definition of genetic groups and representative sample sites for ABC analyses

Motivation

A necessary step prior to conducting any type of ABC analysis is to define genetic groups for use in analyses. If every sample site is used as a genetic group, the number of different possible invasion scenarios becomes computationally prohibitive, and thus this first step is an important one for facilitating coherent analysis (Estoup & Guillemaud 2010; Lombaert *et al.* 2014). Genetic diversity and structure within and between all genotyped sample sites is thus characterized using traditional statistics and clustering methods.

Materials and methods

We genotyped a total of 685 individuals from 23 localities representing the worldwide distribution of *D. suzukii* (table S1 and fig. 1 in main text) at 25 microsatellite loci (Fraimout *et al.* 2015). The complete data file including the multilocus 685 genotypes coded in a 3-digit GENEPOP format (Raymond & Rousset 1995) is available from https://www.researchgate.net/profile/Arnaud_Estoup/contributions. We estimated the level of genetic variability within samples by calculating the mean expected heterozygosity H_e (Nei 1987) and the allelic richness (A) corrected for 15 individuals with FSTAT version 2.9.3.2 (Goudet 2002). We assessed genetic structure between sample sites by computing pairwise F_{ST} values as well as exact test for genotypic differentiation for all pairs of sample sites, using GENEPOP (Raymond & Rousset 1995). Genetic structure was further studied using three complementary clustering methods. First, we used STRUCTURE v2.3.3 (Pritchard *et al.* 2000) assuming a model of admixture with correlated allele frequencies, using parameters set as default and a burn-in period of 10^5 MCMC iterations followed by 10^5 MCMC iterations. The number of possible clusters (K) was set from 1 to 15 and analyses were conducted for 20 replicate runs. Further processing of the STRUCTURE outputs was carried following the methodology described in Lombaert *et al.* (2014) using CLUMP and DISTRUCT softwares (Jakobsson & Rosenberg 2007; Wang *et al.* 2007). Second we used BAPS (Corander *et al.* 2003) analyses carried out on groups of individuals (i.e. sample sites) rather than individuals, with more simple model assumptions (i.e. no admixture and uncorrelated allele frequencies). Third we used the Discriminant Analysis of Principal Components method (DAPC, Jombart *et al.* 2010) implemented in the adegenet R package (v.3.1.0, R core team 2008). DAPC seeks synthetic variables among samples as well as the discriminant functions showing differences between groups as best as possible, while minimizing variation within clusters.

Results

We found substantial polymorphism in our dataset. Heterozygosity was lowest in the invasive Hawaiian sample site (US-Haw, H_e = 0.623) and highest in the native Chinese sample site (CN-Lan, H_e = 0.834; fig. S5). The same populations had the lowest and highest (corrected) allelic diversity ($A_{US\text{-}Haw}$ = 3.392, $A_{CN\text{-}Lan}$ = 9.347). All pairwise comparisons indicated statistically significant genotypic differentiation except for between the two Japanese sample sites and two Chinese sample sites (CN-Lan & CN-Lia; table S5). We found an overall low level of genetic differentiation in the native Asian area with a mean F_{ST} = 0.014 (F_{ST} values between all population pairs are given in table S5). Similarly low levels of

differentiation were found among European populations with a mean F_{ST} of 0.015. We found higher levels of genetic differentiation among continental US populations (mean F_{ST} = 0.044). Eastern US populations showed overall homogeneity (mean F_{ST} = 0.012) whereas the three western US samples revealed a substantially higher level of differentiation (mean F_{ST} = 0.051). North American samples were the least differentiated from the native Asian group (mean F_{ST} = 0.039, between Asia and the western US; mean F_{ST} = 0.056 between Asia and the eastern US) and also showed relatively small differentiation with the Brazilian sample site (mean F_{ST} = 0.028 between the western US and Brazil; mean F_{ST} = 0.031 between the eastern US and Brazil). European sample sites were more differentiated from the native ones (F_{ST} = 0.075) and shown the lowest level of differentiation with La Réunion sample site (mean F_{ST} = 0.046). The Hawaiian sample site showed substantial genetic differentiation with all other samples, with mean F_{ST} values ranging from 0.079 (between Hawaii and the western US) to 0.196 (between Hawaii and La Réunion).

Although genetic clustering analyses using BAPS, STRUCTURE and DAPC did not converge on the same number of genetic groups (K), a strong, shared cluster identity could be observed among clustering methods. The Hawaiian sample site formed a single group in all clustering analyses, consistent with the high F_{ST} values found between Hawaii and all other sample sites. All native Asian sample sites were included in a single cluster in both BAPS and DAPC analyses (fig. 1 in main text and fig. S1, respectively). STRUCTURE results indicated a single cluster in Asia for K = 2 to 5 and Japanese sample sites formed a second Asian cluster for K > 5 (fig. S2). European sample sites systematically formed a single cluster in BAPS, DAPC, and STRUCTURE. A higher heterogeneity in clustering results was observed for North American sample sites. Analyses using BAPS indicated the presence of three genetic groups in North America, which consisted of one eastern US group (including the sample sites US-Col, US-Gen, US-Wis and US-NC), one south-western group (US-Wat and US-SD) and a third group including a single sample site from Oregon state (US-Sok; fig. 1 in main text). Results based on DAPC and STRUCTURE indicated a homogeneous grouping of all sample sites from the eastern US, and distinguished the Oregon sample site (US-Sok) from other continental USA samples, grouping this sample site with Hawaii (fig. S1 and S2, respectively). STRUCTURE analyses showed signatures of genetic introgression from Hawaii in Western US samples, with a stronger introgression pattern in the North than in the South. The Brazilian sample site was assigned along with the eastern US populations by DAPC and STRUCTURE, but to a south-western US group (including the US-Wat and US-SD sample sites) in BAPS. Finally, individuals from La Réunion belonged to the European genetic group in DAPC and STRUCTURE, but formed a single group in BAPS.

We used the results obtained from the three genetic clustering methods along with historical (i.e. dates of first record ; table S1) and geographical information to define seven main genetic groups from our set of 23 sample sites that will be further considered for ABC analyses: Asia (sample sites CN-Lan, CN-Lia, CN-Nin, CN-Shi, JP-Tok and JP-Sap), Hawaii (sample site US-Haw), western US (sample sites US-Wat, US-Sok and US-SD), eastern US (sample sites US-Col, US-NC, US-Wis and US-Gen), Europe (sample sites GE-Dos, FR-Par, FR-Bor, FR-Mon, SW-Del, SP-Bar and IT-Tre), Brazil (BR-PA) and La Réunion (FR-Reu). This allowed us to define eleven nested sets of competing invasion scenarios that we analyzed sequentially using ABC model choice methodologies to reconstruct the worldwide routes of invasion of *D. suzukii* (table 1 in main text).

To reduce computational resources required, ABC treatments were carried out using only one representative sample site for each genetic group per replicate analysis. In other words, when a genetic group included more than two sample sites, we considered the two most differentiated sample sites (i.e. with the highest F_{ST} values in table S5) as representative of the group: JP-Tok and CN-Shi for the group Asia, all three sample sites for the genetically heterogeneous western US group, US-NC and US-Wis for the group eastern US, IT-Tre and GE-Dos for the group Europe

(but see the Results section *Origins of invasive genetic groups* of the main text for a further sub-partitioning of the latter group). Each of the two retained sample sites (i.e. the representative sample sites) were included alternatively in the sample set analyzed using ABC, hence providing the possibility to replicate analyses of scenario choice on different sample sets to verify the robustness of our inferences.

References cited in Appendix S1

Corander J, Waldmann P, Sillanpaa MJ. 2003. Bayesian analysis of genetic differentiation between populations. *Genetics.*163: 367–374.

Estoup A, Guillemaud T. 2010. Reconstructing routes of invasion using genetic data: why, how and so what?. *Mol Ecol.* 19, 4113–4130.

Fraimout A, Loiseau A, Price DK, Xuereb A, Martin JF, Vitalis R, Fellous S, Debat V, Estoup A. 2015. New set of microsatellite markers for the spotted-wing *Drosophila suzukii* (*Diptera: Drosophilidae*): A promising molecular tool for inferring the invasion history of this major insect pest. *Eur J Entomol.* 112(4): 855.

Jakobsson M, Rosenberg NA. 2007. CLUMPP: a cluster matching and permutation program for dealing with label switching and multimodality in analysis of population structure. *Bioinformatics.* 23: 1801–1806.

Jombart T, Devillard S, Balloux F. 2010. Discriminant analysis of principal components: a new method for the analysis of genetically structured populations. *BMC genetics.* 11: 94.

Goudet J. 2002. FSTAT, a program to estimate and test gene diversities and fixation indices (version 2.9.3.2). Updated from Goudet (1995).

Lombaert E, Guillemaud T, Lundgren J, Koch R, Facon B, Grez A, Loomans A, Malausa T, Nedved O, Rhule E, Staverlokk A, Steenberg T, Estoup A. 2014. Complementarity of statistical treatments to reconstruct worldwide routes of invasion: the case of the Asian ladybird *Harmonia axyridis*. *Mol Ecol.* 23: 5979–5997.

Pritchard JK, Stephens M, Donnelly P. 2000. Inference of population structure using multilocus genotype data. *Genetics.*155: 945–959.

Raymond M, Rousset F. 1995. Genepop (version. 1.2), a population genetics software for exact tests and ecumenicism. *J Hered.* 86: 248–249.

Robert CP, Cornuet JM, Marin JM, Pillai NS. 2011. Lack of confidence in approximate

Wang S, Lewis CM, Jakobsson M. *et al.* 2007. Genetic variation and population structure in Native Americans. *Plos Genetics.* 3, 2049–2067.

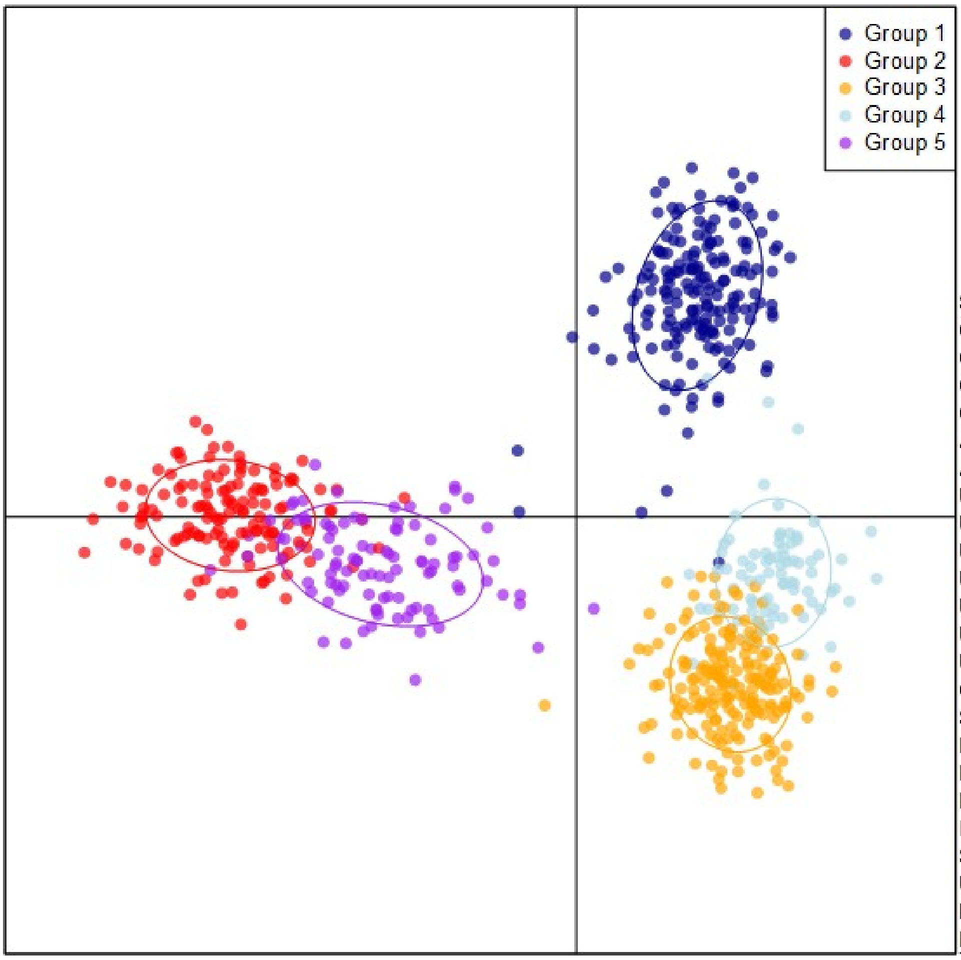

Sample site	Group 1	Group 2	Group 3	Group 4	Group 5
CN-Lan	29	0	0	0	0
CN-Lia	29	0	0	0	0
CN-Shi	30	0	0	0	0
CN-Nin	29	0	0	0	0
JP-Sap	31	0	0	0	0
JP-Tok	30	0	0	0	0
US-Sok	0	0	0	29	0
US-Wat	0	0	11	18	0
US-SD	1	0	37	4	1
US-Col	0	0	28	0	0
US-Wis	0	0	27	0	0
US-Gen	0	0	30	0	0
US-NC	0	0	25	0	0
GE-Dos	0	21	0	0	15
SW-Del	0	19	0	0	10
FR-Par	0	13	0	0	17
FR-Mon	0	16	0	0	11
FR-Bor	0	18	0	0	10
IT-Tre	1	24	0	0	4
SP-Bar	0	17	0	0	13
US-Haw	0	0	0	30	0
BR-PA	0	0	31	0	0
FR-Reu	0	7	0	0	19

Figure S1. Results from analysis using Discriminant Analysis of Principal Components (DAPC). Genetic groups, as inferred by DAPC for each individual, are indicated with different colors. Contributions from all sample sites to each genetic group is detailed in the table at the right, with the number of individuals per sample site assigned to each genetic group. See table S1 and fig. S1 for information on sample sites.

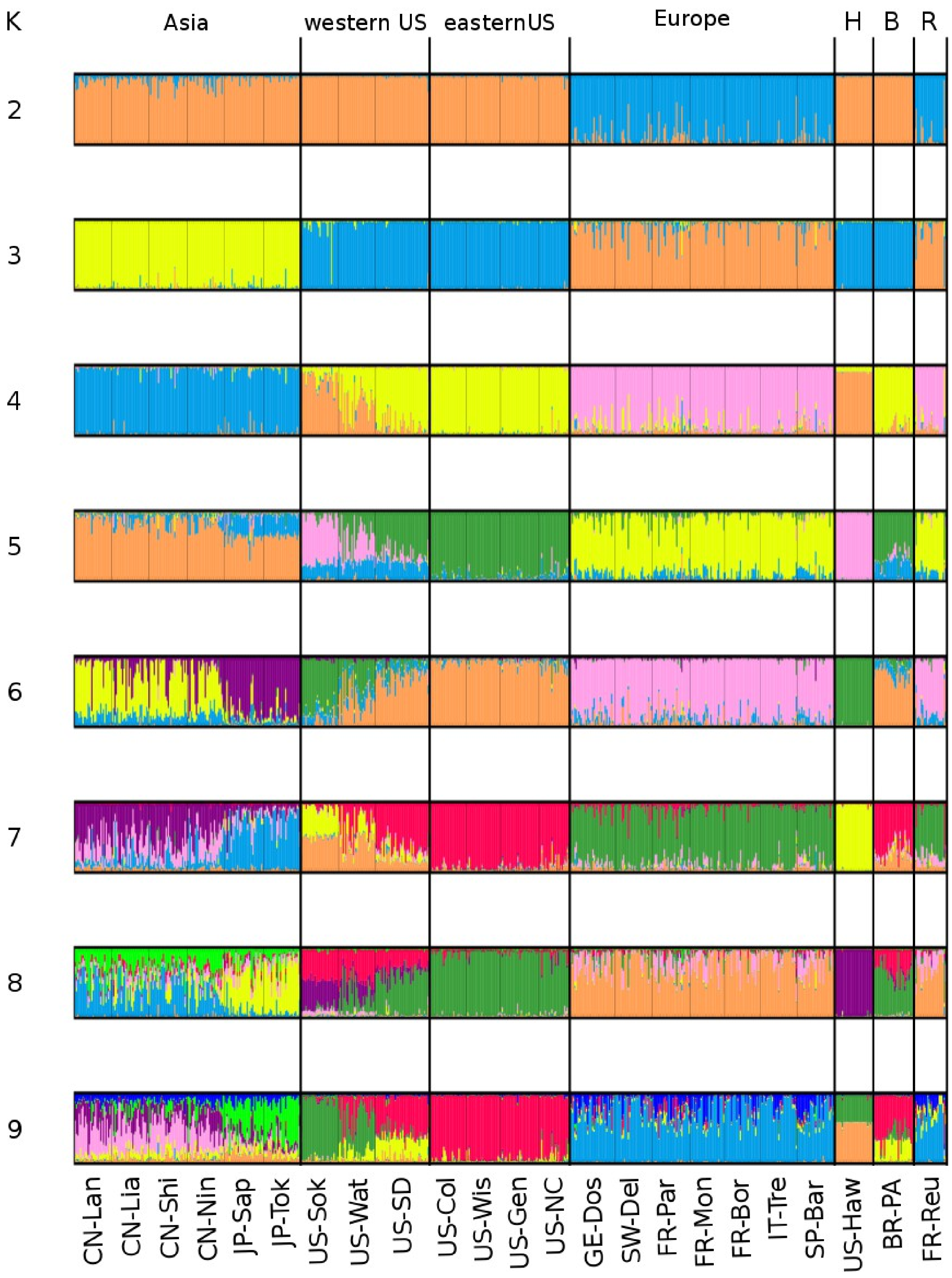

Figure S2. Genetic groups identified by STRUCTURE.

Results are given for a number of genetic group, K, ranging from 2 to 9. Each vertical line represents an individual and each colour represents a genetic group. Individuals are grouped by sample site along the x-

axis. The names at the top of the figure refers to the genetic groups used in ABC analyses. H = Hawaii, B = Brazil, R = La Reunion. The names at the bottom of the figure correspond to the sample sites described in table S1and fig. 1.

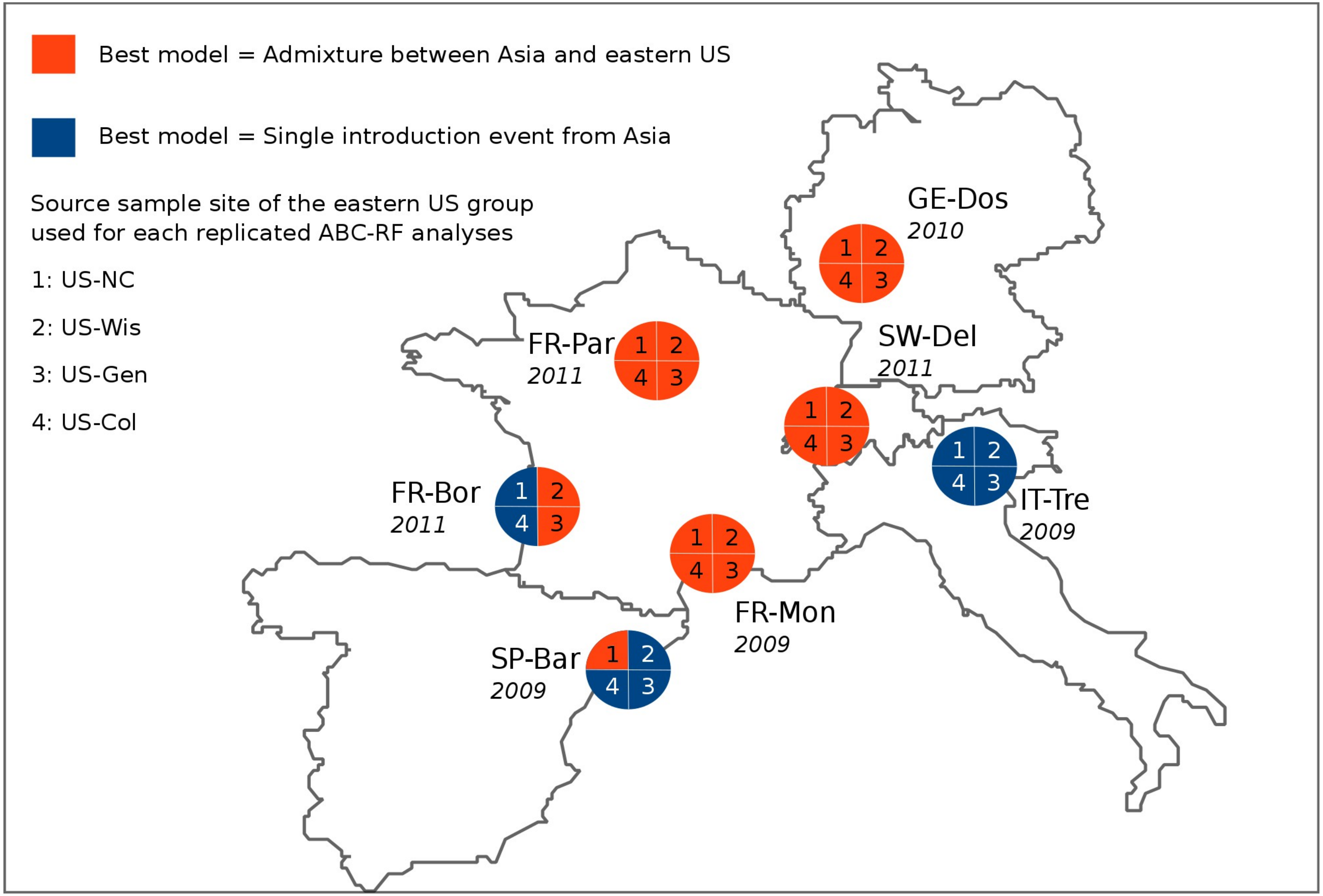
Best model = Admixture between Asia and eastern US
Best model = Single introduction event from Asia
Source sample site of the eastern US group used for each replicated ABC-RF analyses
1: US-NC
2: US-Wis
3: US-Gen
4: US-Col
GE-Dos
2010
FR-Par
2011
SW-Del
2011
FR-Bor
2011
IT-Tre
2009
FR-Mon
2009
SP-Bar
2009
1 2
4 3

Figure S3: Admixed or non-admixed origin of D. suzukii in Europe inferred for each sample sites, using an alternative representative sample site for the native range.

Potential source populations include (among others) the invasive genetic group from eastern US represented by four invasive sample sites (see fig. 1) and the Asian native range. In contrast to fig. 2 of the main text, the Asian native range is here represented by the Chinese sample site CN-Shi, which shows the highest *F*st values with other Asian sample sites (table S3). Four replicate independent ABC-RF treatments corresponding to the analysis 3a (table 1) were carried out for each targeted European sample site using one of the four eastern US sample sites for each treatment (US-NC, US-Wis, US-Gen and US-Col for the treatments labeled 1, 2, 3 and 4 in the pies of the figure, respectively). A pie quarter in blue indicates that the best scenario corresponds to a single introduction event from Asia. A pie quarter in red indicates that the best scenario corresponds to an admixture event between Asia and eastern US.

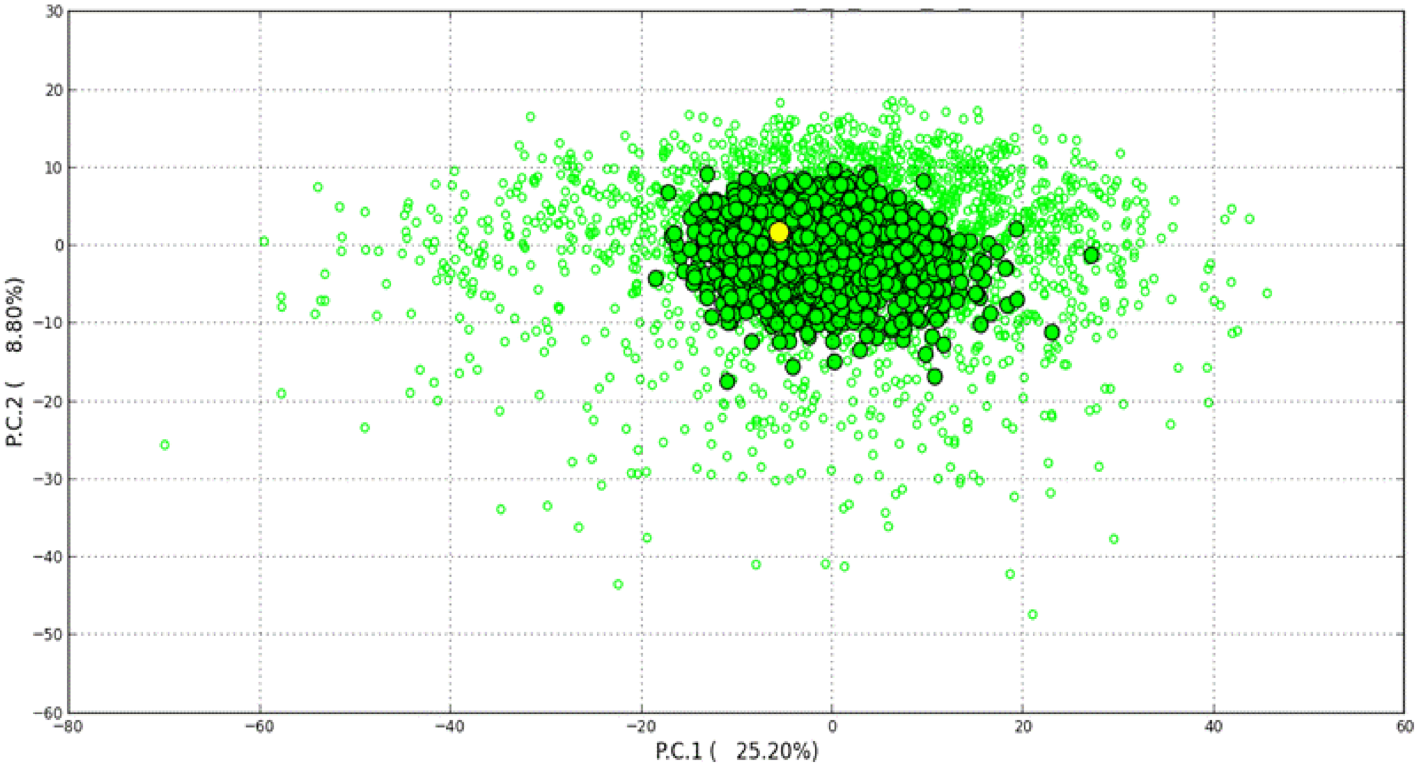

Figure S4. Principal component analysis of 1141 test quantities when processing ABC model-posterior checking for the final worldwide invasion scenario detailed in fig. 1.

The two first axes from the principal component analysis are shown. Small green circles show datasets simulated from priors (subset of 2,000 plots). Large green circles show datasets simulated from posteriors (subset of 2,000 plots). The large yellow circle is the observed dataset. See Materials and methods text section for details.

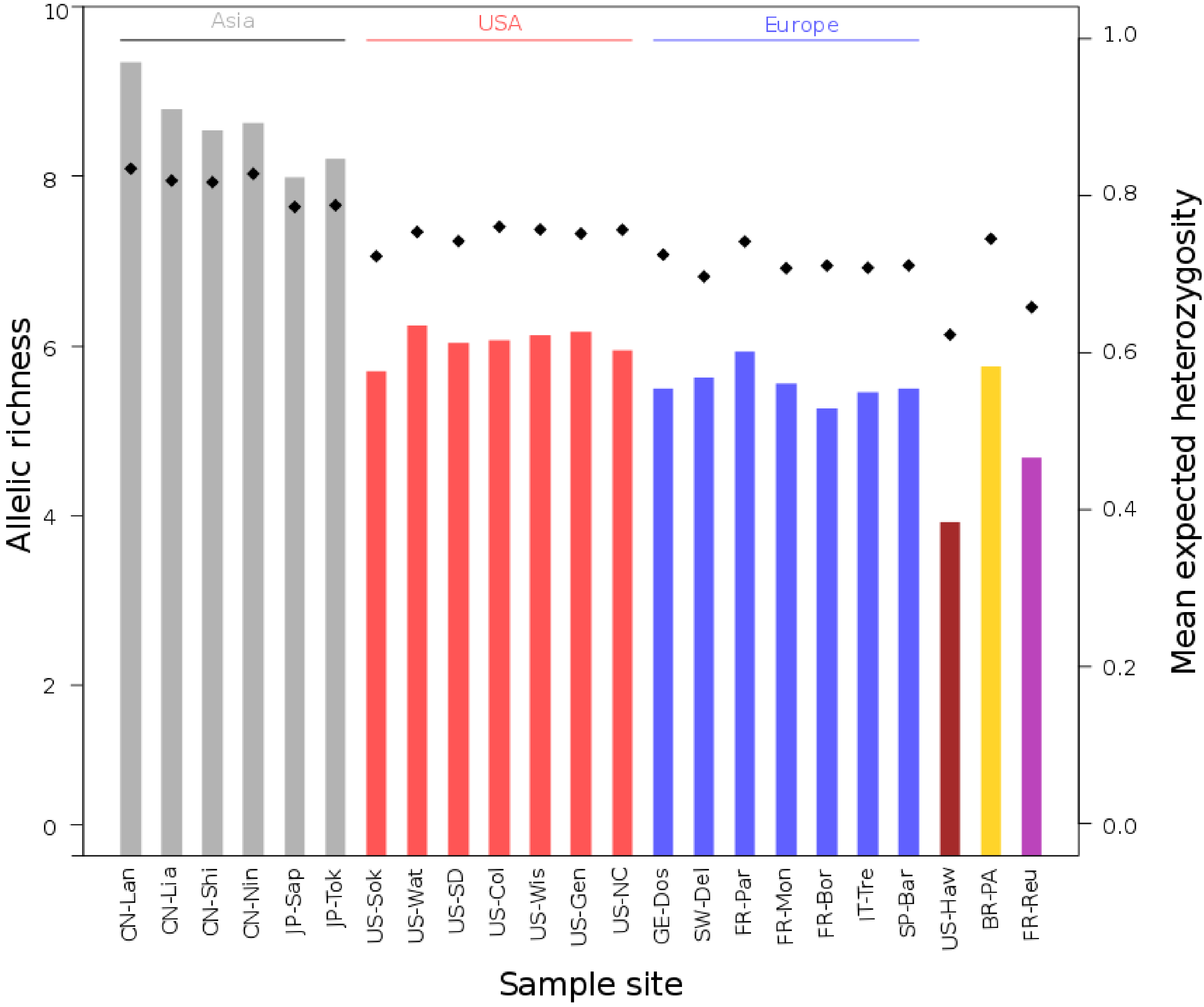

Figure S5. Genetic diversity in the invasive and native sample sites of D. suzukii.

Mean expected heterozygosity (diamonds) and average genetic diversity estimated as allelic richness at 25 microsatellite loci corrected for 15 individuals (bars) are shown. Code names of sample sites given at bottom are the same as those in fig. 1 and table S1.

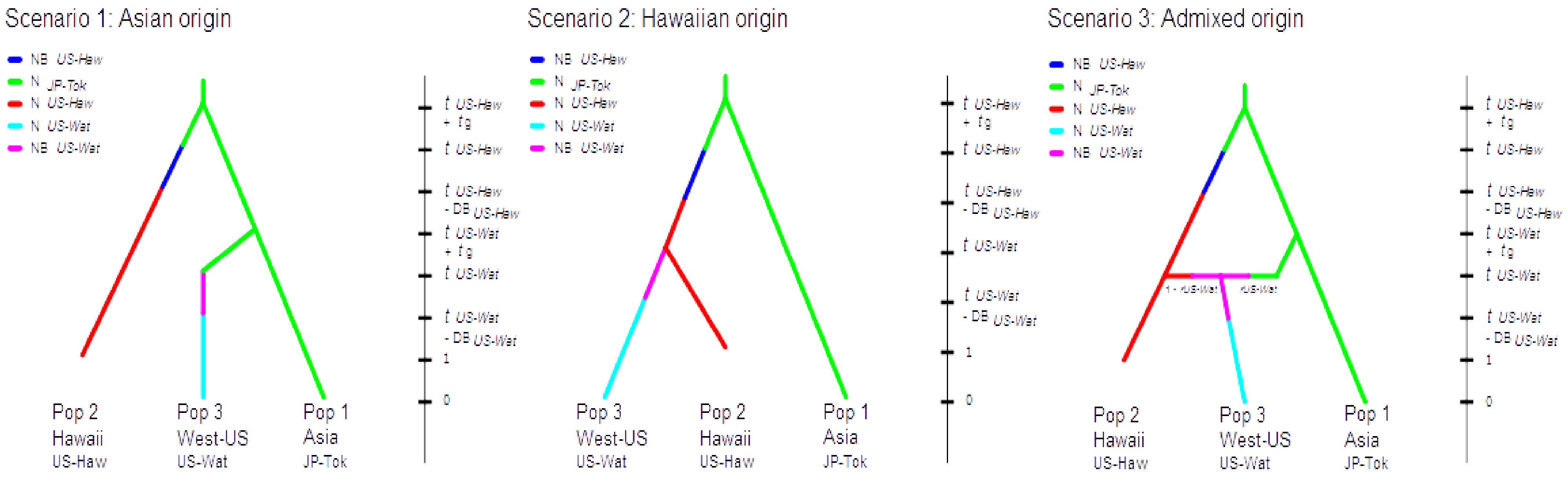

Figure S6. Graphic representation of three ABC scenarios to infer the origin of the US-Wat sample (analysis 1a in table S1 and 2). The western US target genetic unit is represented by the site sample US-Wat and putative sources by US-Haw and JP-Tok (genetic group Hawaii and Asia respectively). Site sample code names are as in table 1 and fig. 1. All parameters with associated prior distributions are described in table S8. Time 0 is the sampling year 2014 and time 1 is the sampling year 2013. Time is not to scale. Scenario 1 corresponds to an Asian native origin (Pop 1) of the target genetic unit (Pop 3). Scenario 2 corresponds to a Hawaiian origin (Pop 2). In scenario 3, the target genetic unit is the result of an admixture between individuals from the Asian group (Pop 1) at a rate $r_{US\text{-}Wat}$ (table 3 and S4) and from the Hawaiian group (Pop 2) at a rate $1 - r_{US\text{-}Wat}$. Note the modeling of a native "ghost population" (i.e., unsampled population) by a green line diverging from Pop 1 at a time $t_{US\text{-}Haw} + t_g$, with t_g drawn into a loose flat prior, which includes zero at the lower bound (table S8)

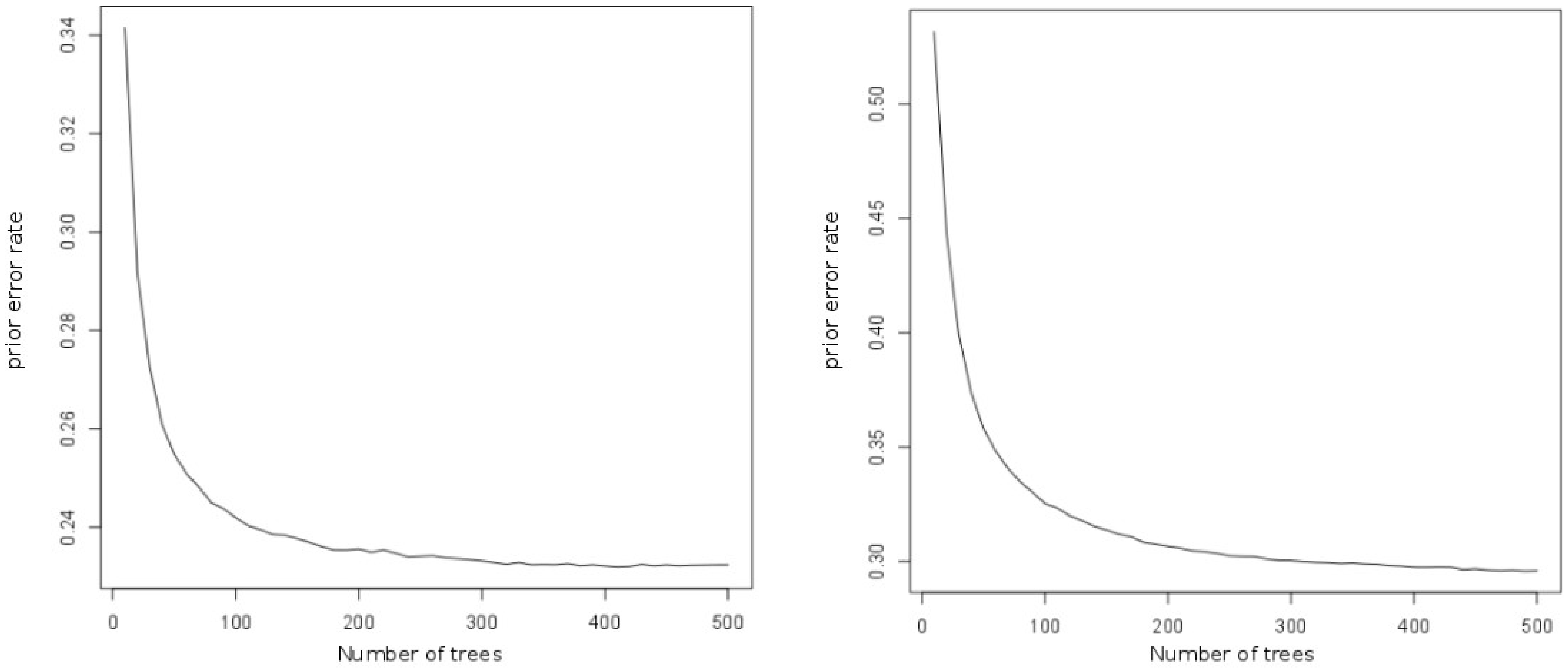

Figure S7. Evolution of the ABC-RF prior error rate with respect to the number of trees in the forest.

Graphs represent the decrease of the ABC-RF prior error rate with the number of trees in the forest for a model setting including six competing scenarios as in analyses 2a and 2b (left pannel) and for a more complex setting including 21 competing scenarios as in analyses 5a and 5b (right pannel).

Sample site name	Historical status	Sampling location	Geographic coordinates	Date of first observation (year)	Sampling year	Number of genotyped individuals
CN-Lan	Native	Langfang city, Hebei province, China	39° 32' 16.969" N 116° 41' 1.507" E	NA	2014	29
CN-Lia	Native	Liaoyuan city, Jilin province, China	42° 53' 15.961" N 125° 8' 37.175" E		2014	29
CN-Shi	Native	Shiping county, Yunnan province, China	23° 42' 21.37" N 102° 29' 41.942 " E		2014	30
CN-Nin	Native	Ningbo city, Zhejiang province, China	29° 52' 6.01" N 121° 32' 38.364" E		2014	29
JP-Tok	Native	Tokyo, Honshu, Japan	35° 41' 22.155" N 139° 41' 30.143" E		2014	30
JP-Sap	Native	Sapporo, Hokkaido, Japan	43° 3' 43.545" N 141° 21' 15.754" E		2014	31
US-Haw	Invasive	Hilo Hawaii, USA	19° 49' 5.305" N 155° 38' 2.743" E	1980	2013	30
US-Wat	Invasive	Watsonville, California, USA	36° 54' 36.832" N 121° 45' 24.82" E	2008	2014	29
US-SD	Invasive	San-Diego, California, USA	32° 42' 56.657" N 117° 9' 39.901" E	2009	2013	43
US-Sok	Invasive	Dayton, Oregon, USA	45° 13' 14.422" N 123° 4' 34.368" E	2009	2014	29
US-NC	Invasive	Raleigh, North Carolina, USA	35° 46' 46.523" N 78° 38' 17.443" E	2010	2014	25
US-Wis	Invasive	Barneveld, Wisconsin, USA	43° 0' 55.989" N 89° 53' 43.443" E	2010	2014	27
US-Gen	Invasive	Geneva, New-York state, USA	42° 52' 4.741" N 76° 59' 9.005" E	2010	2014	30
US-Col	Invasive	Fort Collins, Colorado, USA	40° 35' 6.937" N 105° 5' 3.922" E	2012	2015	28
SP-Bar	Invasive	Barcelona, Spain	41° 23' 6.23" N 2° 10' 24.252" E	2008	2014	30
IT-Tre	Invasive	Trento, Italy	46° 3' 4.321" N 11° 7' 3.141" E	2008	2014	29
FR-Mon	Invasive	Montpellier, France	43° 36' 38.768" N 3° 52' 36.177" E	2009	2013	27
FR-Bor	Invasive	Sauternes, France	44° 31' 51.715" N 0° 20' 35.764" E	2010	2013	28
GE-Dos	Invasive	Dossenheim, Germany	49° 26' 54.02" N 8° 40' 23.714" E	2010	2013	36
FR-Par	Invasive	Paris, France	48° 51' 23.81" N 2° 21' 7.998" E	2010	2015	30
SW-Del	Invasive	Delémont, Switzerland	47° 21' 57.013" N 7° 20' 42.559" E	2011	2013	29
BR-PA	Invasive	Porto Alegre, Brazil	30° 2' 4.763" N 51° 13' 3.57" E	2014	2014	31
FR-Reu	Invasive	La Réunion France	21° 6' 54.508" N 55° 32' 10.982" E	2014	2014	26

Table S1. Locations from which D. suzukii individuals were collected for these analyses, including the date of first observation at the site, and the year sampled.

Analysis	Scenario number	Specifities of the compared scenarios (origin of the focal population)	Focal genetic group
1a+1b+1c	1	Single introduction event from Asia	western US
	2	Single introduction event from Hawaii	
	3	Admixture event between Asia & Hawaii	
1d	1	Single admixture event in US-Wat between Hawaii and Asia, followed by dispersion to US-Sok and US-SD	western US
	2	Single admixture event in US-Wat between Hawaii and Asia, followed by dispersion to US-SD, independent admixture event in US-Sok between Hawaii and Asia	
	3	Single admixture event in US-Wat between Hawaii and Asia, followed by dispersion to US-Sok, independent admixture event in US-SD between Hawaii and Asia	
	4	Three independent admixture events between Hawaii and Asia in each populations	
	5	Single admixture event in US-Wat between Hawaii and Asia, followed by dispersion to US-Sok and US-SD and secondary introduction event from Asia to US-SD	
	6	Single admixture event in US-Wat between Hawaii and Asia, followed by dispersion to US-Sok and US-SD and secondary introduction event from Hawaii in US-Sok	
	7	Single admixture event in US-Wat between Hawaii and Asia, followed by dispersion to US-Sok and US-SD and secondary introduction events from Hawaii in US-Sok and from Asia to US-SD	
2a + 2b	1	Single introduction event from Asia	eastern US (2a)
	2	Single introduction event from Hawaii	or Europe (2b)
	3	Single introduction event from western US	
	4	Admixture event between Asia & Hawaii	
	5	Admixture event between Asia & western US	
	6	Admixture event between Hawaii & western US	
3a + 3b	1	Single introduction event from Asia	eastern US (3a)
	2	Single introduction event from Hawaii	or Europe (3b)
	3	Single introduction event from western US	
	4	Single introduction event from eastern US (3a) or Europe (3b)	
	5	Admixture event between Asia & Hawaii	
	6	Admixture event between Asia & western US	
	7	Admixture event between Asia & eastern US (3a) or Europe (3b)	
	8	Admixture event between Hawaii & western US	
	9	Admixture event between Hawaii &eastern US (3a) or Europe (3b)	
	10	Admixture event between western US & eastern US (3a) or Europe (3b)	
4	1	Admixture event between eastern US & secondary Asian population	northern Europe
	2	Admixture event between eastern US & southern Europe	
5a + 5b	1	Single introduction event from Asia	Brazil (5a)
	2	Single introduction event from Hawaii	or La Réunion (5b)
	3	Single introduction event from western US	
	4	Single introduction event from eastern US	
	5	Single introduction event from southern Europe	
	6	Single introduction event from northern Europe	
	7	Admixture event between Asia & Hawaii	
	8	Admixture event between Asia & western US	
	9	Admixture event between Asia & eastern US	
	10	Admixture event between Asia & southern Europe	
	11	Admixture event between Asia & northern Europe	
	12	Admixture event between Hawaii & western US	
	13	Admixture event between Hawaii & eastern US	
	14	Admixture event between Hawaii & southern Europe	
	15	Admixture event between Hawaii & northern Europe	
	16	Admixture event between western US & eastern US	
	17	Admixture event between western US & southern Europe	
	18	Admixture event between western US & northern Europe	
	19	Admixture event between eastern US & southern Europe	
	20	Admixture event between eastern US & northern Europe	
	21	Admixture event between South and northern Europe	

Table S2. Focal genetic group and origin of its source population(s) for each competing scenarios considered for each eleven ABC analyses.

Note: We formalized competing scenarios in a way that a unique introduction event is defined by a single split from one of the possible sources or in the case of admixture, a split from two different source

populations. See table S1 for information on sample sites including dates of first observation.

			Prior error rate			Posterior probability of the best model		Origin of the focal population using either ABC-RF or ABC-LDA (i.e. best model)
Analysis	Total n° of scenarios	Number of Sum. Stats.	ABC-RF (s.d.)	ABC-LDA (large reference table)	ABC-LDA (small reference table)	ABC-RF (s.d.)	ABC-LDA (large reference table) [CI]	
1a	3	39	0.079 (± 0.001)	0.059	0.080	0.999 (± 0.001)	1.000 [1.000, 1.000]	Asia + Hawaii
1b	3		0.081 (± 0.001)	0.071	0.084	0.999 (± 0.001)	0.999 [0.999, 0.999]	
1c	3		0.077 (± 0.001)	0.062	0.073	1.000 (± 0.000)	0.999 [0.999, 0.999]	
1d	7	130	0.346 (± 0.001)	0.310	0.397	0.665 (± 0.018)	0.612 [0.562, 0.661]	Asia + Hawaii
2a	6	130	0.229 (± 0.001)	0.183	0.269	0.859 (± 0.019)	0.826 [0.802, 0.849]	western US
2b	6	130	0.229 (± 0.001)	0.179	0.305	0.701 (± 0.020)	0.773 [0.760, 0.786]	Asia
3a	10	204	0.350 (± 0.001)	0.314	0.413	0.783 (± 0.016)	0.769 [0.740, 0.797]	western US
3b	10	204	0.354 (± 0.001)	0.341	0.412	0.546 (± 0.023)	0.845 [0.826, 0.864]	Asia
4	2	301	0.156 (± 0.001)	0.217	0.254	0.993 (± 0.004)	0.995 [0.994, 0.996]	southern Europe + eastern US
5a	21	424	0.217 (± 0.001)	0.162	0.417	0.704 (± 0.035)	0.802 [0.769, 0.834]	western US + eastern US
5b	21	424	0.221 (± 0.001)	0.172	0.445	0.594 (± 0.026)	0.292 [0.204, 0.380]	northern Europe + southern Europe

Table S3. Results of model choice analyses using ABC-RF and ABC-LDA, using the prior set 2.
Note: All model choice analyses were carried out considering the prior set 2 (table S8) and a single set of sample sites representative of the pre-defined genetic groups (sample site JP-Tok for the native Asian group, US-Haw for the Hawaiian group, US-Wat, US-Sok and US-SD for the western US group, US-NC for the eastern US group, IT-Tre and GE-Dos for the southern Europe and northern Europe group respectively, BR-PA for the Brazil group and FR-Reu for the La Reunion group; fig. 1). Datasets were summarized using the whole set of summary statistics proposed by the software DIYABC. The total number of summary statistics ("Number of Sum. Stats.") as well as the total number of compared scenarios ("Total n° of scenarios") are indicated for each analysis. Prior error rates and posterior probabilities of the best model chosen using ABC-RF were averaged over ten replicate analyses. ABC-RF and ABC-LDA treatments yielded the same best model

choice for all analyses and is denoted in the column “Origin of the focal population using either ABC-RF or ABC-LDA (i.e. best model)”. Admixture between two source populations are represented by a “+” sign. ABC-LDA posterior probabilities of the best models were estimated using “large reference tables” with 500,000 simulated datasets per scenario. ABC-LDA prior error rates were computed using reference tables of two different sizes: “large reference table” (i.e., 500,000 simulated datasets per scenario) and “small reference table” (i.e., 10,000 simulated datasets per scenario as for ABC-RF analyses). S.d. stands for standard deviation over ten replicate analyses and CI for 95% confidence interval computed following Cornuet *et al.* (2008).

Analysis	Total n° of scenarios	Number of Sum. Stats.	Prior set 1<br>Prior error rate	Posterior probability	Origin of the focal population (best model)	Prior set 2<br>Prior error rate	Posterior probability	Origins of the focal population (best model)
1a	3	39	0.100	1.000	Asia + Hawaii	0.100	1.000	Asia + Hawaii
1b			0.094	0.989		0.094	0.989	
1c			0.096	0.998		0.096	0.998	
1d	7	130	0.327	0.690	Asia + Hawaii	0.327	0.690	Asia + Hawaii
2a	6	130	0.232	0.766	western US	0.227	0.864	western US
2b	6	130	0.224	0.692	Asia	0.224	0.718	Asia + western US
3a	10	204	0.324	0.653	western US	0.355	0.585	western US
3b	10	204	0.393	0.488	Asia + eastern US	0.350	0.501	Asia + eastern US
4	2	301	0.118	1.000	southern Europe + eastern US	0.159	1.000	southern Europe + eastern US
5a	21	424	0.295	0.619	western US + eastern US	0.216	0.674	western US + eastern US
5b	21	424	0.300	0.475	northern Europe + southern Europe	0.223	0.538	northern Europe + southern Europe

Table S4. Results of the ABC-RF analyses defined in table 2 for an alternative set of representative sample sites using prior sets 1 and 2.
Note: The alternative sample sites were chosen considering the most differentiated sites (i.e. sites showing the highest pairwise *F*st value) for each of the ABC groups (see main text and Appendix S1 for details). Here the alternative sample sites are: CN-Shi for Asia, US-Wis for eastern US, GE-Dos for northern Europe and SP-Bar for southern Europe. ABC-RF posterior probabilities of the best model and prior error rates are averaged over three replicate analyses on the same reference table.

**Samples**	CN-Lan	CN-Li	CN-Shi	CN-Nin	JP-Sap	JP-Tok	US-Sok	US-Wat	US-SD	US-Col	US-Wis	US-Gen	US-NC	GE-Dos	SW-Del	FR-Par	FR-Mon	FR-Bor	IT-Tre	SP-Bar	US-Haw	BR-PA
CN-Li	**0.003**																					
CN-Shi	0.014	0.017																				
CN-Nin	0.001	0.002	0.011																			
JP-Sap	0.020	0.017	0.027	0.016																		
JP-Tok	0.022	0.015	**0.032**	0.018	**0.002**																	
US-Sok	0.076	0.071	0.084	0.072	0.060	0.069																
US-Wat	0.054	0.049	0.066	0.049	0.042	0.047	0.026															
US-SD	0.067	0.060	0.081	0.058	0.062	0.067	0.093	0.034														
US-Col	0.074	0.063	0.074	0.067	0.061	0.067	0.095	0.044	0.037													
US-Wis	0.081	0.074	0.085	0.076	0.067	0.077	0.102	0.052	0.046	0.003												
US-Gen	0.071	0.061	0.074	0.063	0.056	0.056	0.097	0.047	0.035	0.009	0.021											
US-NC	0.063	0.054	0.067	0.058	0.052	0.050	0.079	0.034	0.033	0.011	**0.022**	0.005										
GE-Dos	0.072	0.068	0.086	0.068	0.081	0.081	0.135	0.104	0.103	0.107	0.119	0.099	0.092									
SW-Del	0.088	0.090	0.104	0.090	0.108	0.105	0.158	0.135	0.138	0.141	0.145	0.135	0.131	0.023								
FR-Par	0.067	0.063	0.078	0.063	0.083	0.079	0.125	0.094	0.098	0.094	0.109	0.090	0.086	0.019	0.021							
FR-Mon	0.080	0.081	0.100	0.081	0.104	0.096	0.150	0.125	0.126	0.131	0.144	0.125	0.119	0.021	0.015	0.023						
FR-Bor	0.081	0.085	0.099	0.081	0.105	0.101	0.152	0.128	0.133	0.140	0.147	0.134	0.127	0.019	0.008	0.026	0.010					
IT-Tre	0.087	0.088	0.103	0.086	0.111	0.107	0.149	0.132	0.140	0.145	0.152	0.142	0.138	**0.030**	0.005	0.020	0.017	0.009				
SP-Bar	0.077	0.076	0.094	0.075	0.094	0.088	0.141	0.119	0.120	0.128	0.138	0.121	0.115	0.020	0.010	0.015	0.008	0.004	0.003			
US-Haw	0.114	0.124	0.136	0.120	0.103	0.119	0.089	0.090	0.119	0.155	0.161	0.163	0.159	0.169	0.187	0.168	0.184	0.183	0.182	0.169		
BR-PA	0.074	0.068	0.084	0.068	0.071	0.074	0.084	0.031	0.032	0.035	0.041	0.041	0.028	0.115	0.148	0.104	0.132	0.144	0.149	0.135	0.143	
FR-Reu	0.103	0.103	0.118	0.101	0.121	0.120	0.149	0.126	0.137	0.142	0.155	0.127	0.121	0.061	0.059	0.048	0.058	0.056	0.056	0.045	0.197	0.150

Table S5. F_{ST} values between all pair of sample sites.

Note: Code names of the population sites are similar to those in fig.1. Values in blue bold text indicate pairs of samples showing no significant genotypic differentiation. Red bold text indicates the highest F_{ST} value among sample sites within the genetic groups Asia, eastern US and Europe defined using genetic clustering methods along with historical records information (see Appendix S1 for details). See table S1 for information on sample sites.

		Prior 1					Prior 2				
Introduction type	Sample site	mean	median	mode	q5%	q95%	mean	median	mode	q5%	q95%
Extra continental	US-Haw	0.502	0.491	0.481	0.316	0.714	0.482	0.478	0.473	0.374	0.606
	US-Wat	0.171	0.130	0.107	0.039	0.425	0.136	0.119	0.108	0.057	0.264
	IT-Tre	0.204	0.122	0.117	0.034	0.685	0.203	0.159	0.147	0.077	0.496
	BR-PA	0.161	0.129	0.108	0.021	0.405	0.196	0.184	0.167	0.064	0.371
	FR-Reu	0.258	0.211	0.164	0.049	0.628	0.248	0.220	0.186	0.077	0.512
Intra continental	US-SD	0.148	0.135	0.119	0.048	0.284	0.108	0.094	0.083	0.046	0.211
	US-NC	0.124	0.107	0.082	0.033	0.257	0.120	0.108	0.094	0.053	0.225
Extra + intra continental	US-Sok	0.116	0.098	0.086	0.027	0.248	0.112	0.099	0.093	0.048	0.217
	GE-Dos	0.132	0.115	0.081	0.030	0.278	0.162	0.151	0.135	0.069	0.289
Prior values		0.617	0.200	NA	0.020	1.900	0.351	0.200	NA	0.050	0.822

Table S6. Bottleneck severity in invasive populations of D. suzukii for an alternative set of representative sample sites.

Note: The set of population sites used for the ABC estimations presented here include: (i) for the native area: Japan (JP-Tok+JP-Sap), South-East China (CN-Nin) and North-East China (CN-Lan+CN-Lia), and (ii) for the invaded range : US-Wat, US-Sok and US-SD for western US, US-Wis (instead of US-NC) for eastern US, SP-Bar (instead of IT-Tre) for southern Europe, FR-Par (instead of GE-Dos) for northern Europe, BR-PA for South-America (Brazil) and FR-Reu for La Reunion island. Extra-continental introductions correspond to a long distance introduction from a source located apart from the continent of the focal population, intra-continental introduction corresponds to an introduction event from a source located on the same continent than the focal population, and Extra+Intra continental introduction corresponds to a combination of the two types of sources. Mean, median and mode estimates as well as bounds of 90% credibility intervals (q5% and q95%) are indicated for each bottleneck severity parameter. We roughly classified the estimated bottleneck severity values into three classes: weak (i.e. median value of bottleneck severity < 0.12, in light gray), moderate (0.12 < bottleneck severity < 0.22, in gray) and strong (i.e. bottleneck severity > 0.3, in dark gray). Code names of the sample sites are the same as in table S1 and figure 1.

	Admixture event	Admixture rate (gene fraction from pop x)	mean	median	mode	q5%	q95%
Prior 1	A1	r_{US-Wat} (China – O2)	0.763	0.766	0.780	0.662	0.856
	A2	r_{US-Sok} (USA – US-Wat)	0.232	0.226	0.241	0.090	0.393
	A3	r_{FR-Par} (USA – US-Wis)	0.248	0.238	0.216	0.094	0.431
	A4	r_{BR-PA} (USA – SD)	0.689	0.710	0.722	0.420	0.886
	A5	r_{FR-Reu} (Europe – FR-Par)	0.543	0.558	0.599	0.220	0.815
Prior 2	A1	r_{US-Wat} (China – O2)	0.752	0.753	0.756	0.673	0.825
	A2	r_{US-Sok} (USA – US-Wat)	0.222	0.216	0.204	0.111	0.348
	A3	r_{FR-Par} (USA – US-Wis)	0.257	0.248	0.241	0.121	0.415
	A4	r_{BR-PA} (USA – US-SD)	0.620	0.626	0.641	0.434	0.789
	A5	r_{FR-Reu} (Europe – FR-Par)	0.563	0.576	0.606	0.327	0.753

Table S7. Admixture rates estimated for an alternative set of representative sample sites.

Note: Admixture events are denoted as in fig. 1. Each admixture rate parameter *r* points to the name of the admixed population site and corresponds to the fraction of genes originating from the source population site in parentheses (1-*r* genes originate from the other source population site). See table 1 for details on sample sites. Mean, median and mode estimates as well as bounds of 90% credibility intervals (q5% and q95%) are indicated for each admixture parameter. Estimations assuming the prior set 1 and the prior set 2 (table S8) are provided. The alternative set of representative sample sites is the same as the one described in the legend of table S6.

Interpretation	Parameter	Prior distribution set 1	Prior distribution set 2
Stable effective population size	N_i	Uniform [10,000 ; 1,000,000]	Normal [10,000 ; 1,000,000]
Divergence time for the ancestral unsampled "ghost" population	t_{gi}	Uniform [0 ; 20,000]	Normal [25,000 ; 12,500]
Introduction times (in generations)	$t_{US\text{-}Haw}$	Uniform [400 ; 450]	Normal [400 ; 450]
	$t_{US\text{-}Wat,\ SP\text{-}Bar}$	Uniform [80 ; 130]	Normal [80 ; 130]
	$t_{US\text{-}Sok,\ US\text{-}SD,\ IT\text{-}Tre,\ FR\text{-}Mon}$	Uniform [70 ; 120]	Normal [70 ; 120]
	$t_{US\text{-}NC,\ US\text{-}Wis,\ GE\text{-}Dos}$	Uniform [60 ; 110]	Normal [60 ; 110]
	$t_{US\text{-}Gen,\ FR\text{-}Par,\ SW\text{-}Del,\ FR\text{-}Bor}$	Uniform [50 ; 100]	Normal [50 ; 100]
	$t_{US\text{-}Col}$	Uniform [40 ; 90]	Normal [40 ; 90]
	$t_{BR\text{-}PA,\ FR\text{-}Reu}$	Uniform [10 ; 60]	Normal [10 ; 60]
Bottleneck durations	DB_i	Uniform [1 ; 200]	Normal [1 ; 200]
Founding effective population sizes	Nb_i	Uniform [2 ; 1000]	Normal [2 ; 1000]
Admixture rate	r_i	Uniform [0.05 ; 0.95]	Normal [0.500 ; 0.225]
Mutation parameters for	μ	Uniform [1E-6; 1E-5]	LogUniform [1E-6; 1E-5]
microsatellites	P	Uniform [0; 3E-1]	LogUniform [0; 3E-1]
	SNI	Uniform [1E-8; 1E-5]	LogUniform [1E-8; 1E-5]

Table S8. Prior sets 1 and 2: historical, demographic and mutation parameters used for ABC analyses, with their interpretation and their prior distributions.

Note: For a population *i, e*ffective population sizes (N_i) are expressed in number of diploid individuals and times of events (t_i) in number of generations going back

to the past. t_{gi} corresponds to the time of split in number of generations of the unsampled "ghost" population from an ancestral Asian population. Bottleneck durations *DB* for a population *i* indicates the time at which the population will undergo reduction in its effective size from a founding effective population size *NB*. We set the admixture rate *r* as the proportion of genes received by a population *i* from two source populations providing *r* and 1-*r* genes (table 4). The microsatellite loci were assumed to follow a generalized stepwise mutation model with a possible range of 40 contiguous allelic states, and characterized by three parameters: the mean mutation rate (μ), the mean parameter of the geometric distribution of mutation length (*P*), and the mean mutation rate for single nucleotide instability (*SNI*). Each locus is characterized by individual μ_{loc}, P_{loc} and μSNI_{loc} drawn from Gamma distributions. Note that for Uniform [x; y] and logUniform [x; y], x and y are the bounds of the distributions, whereas than for Normal [x; y], x is the mean and y the standard deviation. Prior distribution set 2 was used in order to evaluate the sensitivity of ABC inferences to prior assumptions.

Type of statistics	Summary statistics	Reference
Single sample statistics	Mean number of alleles across loci	
	Mean gene diversity across loci	Nei (1987)
	Mean allele size variance across loci	
	Mean *M* index across loci	Garza & Williamson (2001); Excoffier *et al*. (2005)
Two sample statistics	Mean number of alleles across loci (pooling two samples)	
	Mean gene diversity across loci (pooling two samples)	
	Mean allele size variance across loci (pooling two samples)	
	F_{ST} between two samples	Weir & Cockerham (1984)
	Mean index of classification (relationship between two samples)	Rannala & Moutain (1997); Pascual *et al.* (2007)
	Shared allele distance between two samples	Jin & Chakraborty (1993)
	(δμ)2 distance between two samples	Golstein *et al*. (1995)
Three sample statistics	Maximum likelihood coefficient of admixture (considering one target sample and two source samples)	Choisy *et al*. (2004)

Table S9. Summary statistics used for model choice using ABC-RF and ABC-LDA.

Note: For microsatellite markers, the program DIYABC v.2.1.0 proposes a series of summary statistics among those used by population geneticists. These summary statistics are mean values over loci and characterize a single, a pair or a trio of population samples. More details about such statistics can be found in the following list of references:

Choisy M, Franck P, Cornuet JM. 2004. Estimating admixture proportions with microsatellites: comparison of methods based on simulated data. *Mol Ecol*. 13: 955 –

968.

Excoffier L, Estoup A, Cornuet JM. 2005. Bayesian analysis of an admixture model with mutations and arbitrarily linked markers. *Genetics* 169: 1727-1738.

Garza JC, Williamson E. 2001. Detection of reduction in population size using data from microsatellite DNA. *Mol Ecol.* 10: 305-318.

Goldstein DB, Linares AR, Cavalli-Sforza LL, Feldman MW. 1995. An evaluation of genetic distances for use with microsatellite loci. *Genetics* 139: 463-471.

Jin L, Chakraborty R. 1993. Estimation of genetic distance and coefficient of gene diversity from single-probe multilocus DNA fingerprinting data. *Mol Biol Evol*. 11:120-127.

Nei M. 1987. Molecular Evolutionary Genetics. *New York: Columbia University Press.*

Pascual M, Chapuis M-P, Mestres F, Balany J, Huey RB, Gilchrist GW, L. Serra L, Estoup A. 2007. Introduction history of *Drosophila subobscura* in the New World : a microsatellite based survey using ABC methods. *Mol Ecol.* 16: 3069-3083.

Rannala B, Mountain JL. 1997. Detecting immigration by using multilocus genotypes. *Proc. Nat. Acad. Sci. USA.* 94: 9197-9201.

Weir BS, Cockerham CC. 1984. Estimating F-statistics for the analysis of population structure. *Evolution* 38: 1358-1370.

Appendix S2: R scripts to run ABC-RF model choice analyses using datasets simulated with the package DIYABC v2.1.0

Motivation

For all ABC-RF model choice analyses, we used the software DIYABC v.2.1.0 (Cornuet et al. 2014) to simulate datasets constituting the reference tables. A reference table includes a given number of datasets that have been simulated for different scenarios using parameter values drawn from prior distributions, each dataset being summarized with a pool of statistics. Typically, DIYABC v2.1.0 produces a binary file, named reftable.bin, corresponding to the reference table. Following Pudlo et al. (2016), random forest treatments were processed on such reference tables (typically including 10,000 simulated datasets per scenario) using the abcrf R package (v1.1.0; Pudlo et al. 2016). Note that the linear discriminant analysis (LDA) axes used as additional summary statistics in the random forest analysis are automatically computed and added to the summary statistics by the program abcrf.

In this appendix, we provide three thoroughly commented (home-made) scripts written in R programming language (R Development Core Team 2008). These new scripts represent useful code resources for computing random forest analyses in R with the abcrf package, when starting from simulated datasets generated with the widely used ABC package DIYABC v.2.1.0.

Script #1: this basic R script allows to (i) running a single ABC-RF model choice analysis from a single dataset of observed summary statistics starting from a DIYABC v2.1.0 reference table and (ii) obtain some detailed numerical outputs and several graphical illustrations of analysis result.

```
# R SCRIPT#1 to run a single ABC-RF model choice analysis starting from the reference table generated by the DIYABC v2.1.0 package (.bin format) and from a single dataset of observed summary statistics
# This script includes some code to obtain some detailed numerical outputs and several graphical illustrations of analysis results
# Date: 01/09/2016 version 1.0.
# Licence: GPL2
# Authors: Arnaud Estoup, Jean-Michel Marin, Julien Foucaud, Alex Dehne-Garcia, and Antoine Fraimout

# Note: the example below is based on a DIYABC reference table named "reftable.bin" including 4 scenarios (models) and a total of 40000 simulated datasets summarized with 130 summary statistics

# Preliminary steps:

# STEP1 - Install the R package abcrf (Pudlo P, Marin JM, Estoup A, Cornuet JM, Gautier M, Robert CP. 2016. Reliable ABC model choice via random forests. Bioinformatics. 32: 859-866.)
# Load and install automatically the package "abcrf" from the CRAN by clicking in the corresponding option of a R_Gui or R_Studio console (option "Packages" in RGui and "Intall Packages" in RStudio")
install.packages("abcrf")
# or if you got the abcrf source file then write: install.packages("abcrf_1.1.tar.gz", repos = NULL, type="source")
library(abcrf)

# STEP2 - Go into your working directory which may be the DIYABC project directory.
setwd("/pathway_for_my_working_directory/")
# At minimum the chosen working directory should contain the following DIYABC files:
# the reference table "reftable.bin" (generated with DIYABC v2.1.0), the file RNG_state_0000.bin and the datafile "statobs.txt" (the latter including the summary statistics of the observed dataset)
# Add in the working directory the DIYABC v2.1.0 core executable file which can be downloaded at http://www1.montpellier.inra.fr/CBGP/diyabc/ for different operating systems (cf. downloadable file diyabc_core-2.1.0-linWinOsXExe.zip).
# The name of the DIYABC 2.1 core executable files is:
# diyabc_core-2.1.0-win.exe for windows
# diyabc_core-2.1.0-OsX for mac
# diyabc_core-2.1.0-linux-i386 or diyabc_core-2.1.0-linux-x64 or diyabc_core-2.1.0-OldLinux-x64 (or the executable file obtained through your own compilation) for linux
```

```
# STEP 3 - Convert the reftable.bin file into a reftable.txt file (cf. reference table file with a txt format) that will be ready
to use by the present R script
# To do that, open a terminal, go to your working directory and write the following instruction:
# In a linux OS write: ./ diyabc_core-2.1.0-linux-i386 -p ./ -x
# In a windows OS write: diyabc_core-2.1.0-win.exe -p ./ -x
# In a mac OS write: ./ diyabc_core-2.1.0-OsX -p ./ -x

# Definition of the key parameters of the RF analysis:

# Number of compared scenarios (models) in the reference table
nscenarios=4
# Number of summary statistics in the reference table
nSS=130
# Size of the reference table (i.e. number of simulated dataset) that will be used to do the RF analysis
Nref= 40000
# Number of trees in the random forest (default = 500)
ntrees_in_forest=500

# Reading of the DIYABC datafile "statobs.txt" (the latter including the summary statistics of the observed dataset)
#  Note: Usually the file statobs.txt includes a single row corresponding to a single vector of observed summary statistics
# see the R script # 3 for the treatment of a statobs.txt file including several rows corresponding to several vectors of
observed summary statistics
statobs=read.table("statobs.txt",header=TRUE)

# Procedure to read the file reftable.txt and store data in a correct way for RF analysis
# Note: The procedure is a bit complex as it allows dealing with DIYABC reftables in which the number of parameters
may be different for the different scenarios
# T1 = starting time of the analysis (including reading the file reftable.txt)
T1<-Sys.time()
ncol=max(count.fields("reftable.txt",skip=2*nscenarios+3))
toto=read.table("reftable.txt",skip=2*nscenarios+3,header=FALSE,fill=TRUE,row.names=NULL,col.names=paste("V",1:
ncol,sep=""),nrows=Nref)
toto=toto[order(toto[,1]),]
rm(ncol)
import.reftable=function(nb.SS=nSS)
{
scenarios=unique(toto[,1])
sortie=data.frame()
param=list()
long=rep(0,length(scenarios))
for(scen in scenarios)
{
lignes.scen=which(toto[,1]==scen)
nb.col=ncol(toto[lignes.scen[1],])-sum(is.na(toto[lignes.scen[1], ]))
extrait=toto[lignes.scen,1:nb.col]
param0=extrait[, 2:(nb.col-nb.SS)]
long[scen]=ncol(param0)
extrait=extrait[,c(1, (nb.col - nb.SS + 1):nb.col)]
colnames(extrait)=1:(nb.SS+1)
colnames(param0)=1:(ncol(param0))
rownames(extrait)=rownames(param0)=NULL
sortie=rbind(sortie,extrait)
param[[scen]]=param0
}
max.param.dim=max(long)
for(scen in scenarios)
{
if(ncol(param[[scen]])<max.param.dim)
param[[scen]]=cbind(param[[scen]],matrix(NA,nrow=nrow(param[[scen]]),ncol=max.param.dim-ncol(param[[scen]])))
}
```

```
mes.param=data.frame()
for(scen in scenarios)
{
colnames(param[[scen]])=1:max.param.dim
rownames(param[[scen]])=NULL
mes.param=rbind(mes.param, as.matrix(param[[scen]]))
}
return(list(ss=sortie, param=mes.param))
}
tmp=import.reftable()
indi=sample(1:nrow(tmp$ss),Nref)
reftable=tmp$ss[indi,]
param=tmp$param[indi,]

# Further tuning of the structure of reftable
colnames(reftable) <- c("modindex", colnames(statobs))
modindex <- as.factor(reftable[1:Nref,1])
sumsta <- reftable[1:Nref,-1]

### WE CAN NOW START RANDOM FOREST COMPUTATIONS #########
# Computation related to the forest classification
mc.rf <- abcrf(modindex,sumsta,paral=TRUE,ntree=ntrees_in_forest)
## Computation to identify the best model for the observed dataset and estimate its posterior probability
# The function "predict" provides the ID of the selected (i.e. best) model (scenario), the votes for each compared scenarios (over ntrees_in_forest),
# knowing that the best scenario is the one with the highest vote value, and the value of the posterior probability of the best model
predict(mc.rf,statobs,paral=TRUE,ntree=ntrees_in_forest)

# Duration of the analysis (including reading the file reftable.txt)
T2<-Sys.time()
duration = difftime(T2, T1)
duration

### CODE TO VISUALIZE RESULTS AND VARIOUS GRAPHICAL ILLUSTRATIONS################

# Visualize numerical results for the global prior error rates and the matrix of confusion (i.e. prior error rates detailed for each scenario)
# Estimation from 10000 pseudo-observed datasets from the reference table
mc.rf

# Vizualize and save in pdf files several useful illustrative graphics

# Graphic providing prior error rates for forests with different number of trees (pdf file name = ""error_vs_ntree.pdf")
# e.g. Fig. 3 in Pudlo et al. 2016
err.rf <- err.abcrf(mc.rf)
x11()
plot(err.rf)
pdf(file="error_vs_ntree.pdf",h=18,w=18)
plot(err.rf)
dev.off()

# Two graphics/figures providing (i) LDA projections of the reference table for the different scenarios plus the observed dataset (cf. black star in the figure)
# (file named graph_lda.pdf) and (ii) the contributions of the 30 most important statistics to the RF (file named graph_varImpPlot.pdf)
# e.g. Fig. S6 and Fig. S7 in Pudlo et al. 2016
plot(mc.rf,statobs,pdf=TRUE,n.var=30)
```

Script #2: this second R script allows to running successively several ABC-RF model choice analyses starting from

the same DIYABC v2.1.0 reference table and this for single dataset of observed summary statistics

```
# R SCRIPT#2 to run successively SEVERAL ABC-RF model choice analyses starting from the SAME reference table generated by the DIYABC v2.1.0 package (.bin format) and from a single dataset of observed summary statistics
# Date: 01/09/2016 version 1.0.
# Licence: GPL2
# Authors: Arnaud Estoup, Jean-Michel Marin, Julien Foucaud, Alex Dehne-Garcia, and Antoine Fraimout
# Note: the example below is based on a DIYABC reference table named "reftable.bin" including 4 scenarios (models) and a total of 40000 simulated datasets summarized with 130 summary statistics
# One wants 3 successive ABC-RF model choice analyses from a given reference table: the first one with 20000 simulated datasets, the second one with 30000 simulated datasets and third one with 40000 simulated datasets.

# Preliminary steps:

# STEP1 - Install the R package abcrf (Pudlo P, Marin JM, Estoup A, Cornuet JM, Gautier M, Robert CP. 2016. Reliable ABC model choice via random forests. Bioinformatics. 32: 859-866.)
# Load and install automatically the package "abcrf" from the CRAN by clicking in the corresponding option of a R_Gui or R_Studio console (option "Packages" in RGui and "Intall Packages" in RStudio")
install.packages("abcrf")
# or if you got the abcrf source files then write: install.packages(“abcrf_1.1.tar.gz”, repos = NULL, type="source")
library(abcrf)

# STEP2 - Go into your working directory which may be the DIYABC project directory.
setwd("/pathway_for_my_working_directory/")
# At minimum the chosen working directory should contain the following DIYABC files:
# the reference table "reftable.bin" (generated with DIYABC v2.1.0), the file RNG_state_0000.bin and the datafile "statobs.txt" (the latter including the summary statistics of the observed dataset)
# Add in the working directory the DIYABC v2.1.0 core executable file which can be downloaded at http://www1.montpellier.inra.fr/CBGP/diyabc/ for different operating systems (cf. downloadable file diyabc_core-2.1.0-linWinOsXExe.zip).
# The name of the DIYABC 2.1 core executable files is:
# diyabc_core-2.1.0-win.exe for windows
# diyabc_core-2.1.0-OsX for mac
# diyabc_core-2.1.0-linux-i386 or diyabc_core-2.1.0-linux-x64 or diyabc_core-2.1.0-OldLinux-x64 (or the executable file obtained through your own compilation) for linux

# STEP 3 - Convert the reftable.bin file into a reftable.txt file (cf. reference table file with a txt format) that will be ready to use by the present R script
# To do that, open a terminal, go to your working directory and write the following instruction:
# In a linux OS write: ./ diyabc_core-2.1.0-linux-i386 -p ./ -x
# In a windows OS write: diyabc_core-2.1.0-win.exe -p ./ -x
# In a mac OS write: ./ diyabc_core-2.1.0-OsX -p ./ -x

# Definition of the key parameters of the RF analysis:

# Number of compared scenarios (models) in the reference table
nscenarios=4
# Number of summary statistics in the reference table
nSS=130
# Size of the reference table (i.e. number of simulated dataset) that will be used to do the successive RF analyses
Nrefmax=40000
# Vector defining the 3 successive ABC-RF model choice analyses from a given reference table a size Nrefmax: the third one with 20000 simulated datasets,
# the second one with 30000 simulated datasets and first one with 40000 simulated datasets.
Nref= c(20000,30000,40000)
# Number of trees in the random forest (default = 500)
ntrees_in_forest=500

# Name and structure of the output file that will summarize numerical results for all successive RF analyses
```

```
# Outputs for each analysis are presented as following: "Nref best_scenario posterior_probability vote_scen1 vote_scen2 vote_scen3 vote_scen4 prior_error_rate
output_file_name <- "output_RF.txt"
output <- data.frame(matrix(ncol=nscenarios+4))
options(digits=4)

# Reading of the DIYABC datafile "statobs.txt" (the latter including the summary statistics of the observed dataset)
#  Note: Usually the file statobs.txt includes a single row corresponding to a single vector of observed summary statistics
# see the R script # 3 for the treatment of a statobs.txt file including several rows corresponding to several vectors of observed summary statistics
statobs=read.table("statobs.txt",header=TRUE)

# Procedure to read the file reftable.txt and store data in a correct way for RF analyses
# Note: The procedure is a bit complex as it allows dealing with DIYABC reftables in which the number of parameters may be different for the different scenarios
# T1 = starting time of the analysis (including reading the file reftable.txt)
T1<-Sys.time()
ncol=max(count.fields("reftable.txt",skip=2*nscenarios+3))
toto=read.table("reftable.txt",skip=2*nscenarios+3,header=FALSE,fill=TRUE,row.names=NULL,col.names=paste("V",1:ncol,sep=""),nrows=Nrefmax)
toto=toto[order(toto[,1]),]
rm(ncol)
import.reftable=function(nb.SS=nSS)
{
scenarios=unique(toto[,1])
sortie=data.frame()
param=list()
long=rep(0,length(scenarios))
for(scen in scenarios)
{
lignes.scen=which(toto[,1]==scen)
nb.col=ncol(toto[lignes.scen[1],])-sum(is.na(toto[lignes.scen[1], ]))
extrait=toto[lignes.scen,1:nb.col]
param0=extrait[, 2:(nb.col-nb.SS)]
long[scen]=ncol(param0)
extrait=extrait[,c(1, (nb.col - nb.SS + 1):nb.col)]
colnames(extrait)=1:(nb.SS+1)
colnames(param0)=1:(ncol(param0))
rownames(extrait)=rownames(param0)=NULL
sortie=rbind(sortie,extrait)
param[[scen]]=param0
}
max.param.dim=max(long)
for(scen in scenarios)
{
if(ncol(param[[scen]])<max.param.dim)
param[[scen]]=cbind(param[[scen]],matrix(NA,nrow=nrow(param[[scen]]),ncol=max.param.dim-ncol(param[[scen]])))
}
mes.param=data.frame()
for(scen in scenarios)
{
colnames(param[[scen]])=1:max.param.dim
rownames(param[[scen]])=NULL
mes.param=rbind(mes.param, as.matrix(param[[scen]]))
}
return(list(ss=sortie, param=mes.param))
}
tmp=import.reftable()

########## STARTING SERIAL ABCRF COMPUTATIONS #################
```

```
for (i in 1:length(Nref))
{
	indi=sample(1:nrow(tmp$ss),Nref[i])
	reftable=tmp$ss[indi,]
	param=tmp$param[indi,]
	colnames(reftable) <- c("modindex", colnames(statobs))
	modindex <- as.factor(reftable[1:Nref[i],1])
	sumsta <- reftable[1:Nref[i],-1]
	mc.rf <- abcrf(modindex,sumsta,paral=TRUE,ntrees_in_forest)
	pred.rf <- predict(mc.rf,statobs,paral=TRUE,ntrees_in_forest)
	output <- rbind(output, c(Nref[i], as.numeric(pred.rf[1]),
		round(as.numeric(pred.rf[length(pred.rf)]),4),
		unlist(pred.rf[2:(length(pred.rf)-1)]),
		round(as.numeric(mc.rf$prior.err),4)))
}

output <- output[-1,]
colnames(output) <- c("Nref", "Best_scenario", "Posterior_probability", rownames(as.data.frame(unlist(pred.rf[2:
(length(pred.rf)-1)]))), "Prior_error_rate")
write.table(output, output_file_name, quote=FALSE, row.names=FALSE)

# Duration of the analysis (including reading the file reftable.txt)
T2<-Sys.time()
duration = difftime(T2,T1)
duration

rm(list=ls())

# END OF COMPUTATION: Open and edit the output file (output_file_name as defined above) which contains the
numerical results of the ABC RF analyses: for each analysis outputs are presented as following: Nref Statobs_row_ID
Best_scenario Posterior_probability vote_scen1 vote_scen2 vote_scen3 vote_scen4 Prior_error_rate
```

Script #3: this third R script allows to running successively several ABC-RF model choice analyses starting from the same DIYABC v2.1.0 reference table and this for a data file including several observed datasets.

```
# R SCRIPT#3 to run successively SEVERAL ABC-RF model choice analyses starting from the SAME reference table
generated by the DIYABC v2.1.0 package (.bin format)
# and this for a data file including SEVERAL rows corresponding to several vectors of observed summary statistics.
# Date: 01/09/2016 version 1.0.
# Licence: GPL2
# Authors: Arnaud Estoup, Jean-Michel Marin, Julien Foucaud, Alex Dehne-Garcia, and Antoine Fraimout
# Note: the example below is based on a DIYABC reference table named "reftable.bin" including 4 scenarios (models)
and a total of 40000 simulated datasets summarized with 130 summary statistics
# One wants 6 successive ABC-RF model choice analyses from a given reference table: the first one with 20000
simulated datasets, the second one with 30000 simulated datasets and third one with 40000 simulated datasets;
# each of the three analyses are processed on 3 vectors of observed summary statistic (in the statobs.txt data file); hence a
total of 6 RF analyses.
# Notice that the (observed) data file may include much larger number of vectors of observed summary statistic without
increasing too much the RF computational cost.

# Preliminary steps:

# STEP1 - Install the R package abcrf (Pudlo P, Marin JM, Estoup A, Cornuet JM, Gautier M, Robert CP. 2016. Reliable
ABC model choice via random forests. Bioinformatics. 32: 859-866.)
# Load and install automatically the package "abcrf" from the CRAN by clicking in the corresponding option of a R_Gui
or R_Studio console (option "Packages" in RGui and "Intall Packages" in RStudio")
install.packages("abcrf")
# or if you got the abcrf sources file then write: install.packages("abcrf_1.1.tar.gz", repos = NULL, type="source")
```

```
library(abcrf)

# STEP2 - Go into your working directory which may be the DIYABC project directory.
setwd("/pathway_for_my_working_directory/")
# At minimum the chosen working directory should contain the following DIYABC files:
# the reference table "reftable.bin" (generated with DIYABC v2.1.0), the file RNG_state_0000.bin and the datafile "statobs.txt" (the latter including the summary statistics of the observed dataset)
# Add in the working directory the DIYABC v2.1.0 core executable file which can be downloaded at http://www1.montpellier.inra.fr/CBGP/diyabc/ for different operating systems (cf. downloadable file diyabc_core-2.1.0-linWinOsXExe.zip).
# The name of the DIYABC 2.1 core executable files is:
# diyabc_core-2.1.0-win.exe for windows
# diyabc_core-2.1.0-OsX for mac
# diyabc_core-2.1.0-linux-i386 or diyabc_core-2.1.0-linux-x64 or diyabc_core-2.1.0-OldLinux-x64 (or the executable file obtained through your own compilation) for linux

# STEP 3 - Convert the reftable.bin file into a reftable.txt file (cf. reference table file with a txt format) that will be ready to use by the present R script
# To do that, open a terminal, go to your working directory and write the following instruction:
# In a linux OS write: ./ diyabc_core-2.1.0-linux-i386 -p ./ -x
# In a windows OS write: diyabc_core-2.1.0-win.exe -p ./ -x
# In a mac OS write: ./ diyabc_core-2.1.0-OsX -p ./ -x

# Definition of the key parameters of the RF analysis:

# Number of compared scenarios (models) in the reference table
nscenarios=4
# Number of summary statistics in the reference table
nSS=130
# Size of the reference table (i.e. number of simulated dataset) that will be used to do the successive RF analyses
Nrefmax=40000
# Vector defining the 3 successive ABC-RF model choice analyses from a given reference table a size Nrefmax: the third one with 20000 simulated datasets,
# the second one with 30000 simulated datasets and first one with 40000 simulated datasets.
Nref= c(20000,30000,40000)
# Number of trees in the random forest (default = 500)
ntrees_in_forest=500

# Name and structure of the output file that will summarize numerical results for all successive RF analyses
# Outputs for each analysis are presented as following: Nref Statobs_row_ID Best_scenario Posterior_probability vote_scen1 vote_scen2 vote_scen3 vote_scen4 Prior_error_rate
output_file_name <- "output_RF.txt"
output <- data.frame(matrix(ncol=nscenarios+5))
options(digits=4)

# Reading of the DIYABC datafile "statobs.txt" (the latter including the summary statistics of the observed dataset(s))
#  Here statobs.txt file includes 3 rows corresponding to 3 vectors of observed summary statistics that will be analysed serially.
# You can add a text identifier at the beginning of each vectors of observed summary statistics of the DIYABC datafile initially named "statobs.txt" and now renamed "statobs_multi.txt" (for instance "dataobs_case_x"): this additional text will not hinder the analyses
statobs=read.table("statobs_multi.txt",header=TRUE)

# Procedure to read the file reftable.txt and store data in a correct way for RF analyses
# Note: The procedure is a bit complex as it allows dealing with DIYABC reftables in which the number of parameters may be different for the different scenarios
# T1 = starting time of the analysis (including reading the file reftable.txt)
T1<-Sys.time()
ncol=max(count.fields("reftable.txt",skip=2*nscenarios+3))
```

```
toto=read.table("reftable.txt",skip=2*nscenarios+3,header=FALSE,fill=TRUE,row.names=NULL,col.names=paste("V",1:
ncol,sep=""),nrows=Nrefmax)
toto=toto[order(toto[,1]),]
rm(ncol)
import.reftable=function(nb.SS=nSS)
{
scenarios=unique(toto[,1])
sortie=data.frame()
param=list()
long=rep(0,length(scenarios))
for(scen in scenarios)
{
lignes.scen=which(toto[,1]==scen)
nb.col=ncol(toto[lignes.scen[1],])-sum(is.na(toto[lignes.scen[1], ]))
extrait=toto[lignes.scen,1:nb.col]
param0=extrait[, 2:(nb.col-nb.SS)]
long[scen]=ncol(param0)
extrait=extrait[,c(1, (nb.col - nb.SS + 1):nb.col)]
colnames(extrait)=1:(nb.SS+1)
colnames(param0)=1:(ncol(param0))
rownames(extrait)=rownames(param0)=NULL
sortie=rbind(sortie,extrait)
param[[scen]]=param0
}
max.param.dim=max(long)
for(scen in scenarios)
{
if(ncol(param[[scen]])<max.param.dim)
param[[scen]]=cbind(param[[scen]],matrix(NA,nrow=nrow(param[[scen]]),ncol=max.param.dim-ncol(param[[scen]])))
}
mes.param=data.frame()
for(scen in scenarios)
{
colnames(param[[scen]])=1:max.param.dim
rownames(param[[scen]])=NULL
mes.param=rbind(mes.param, as.matrix(param[[scen]]))
}
return(list(ss=sortie, param=mes.param))
}
tmp=import.reftable()

########## STARTING SERIAL ABCRF COMPUTATIONS #################
for (i in 1:length(Nref)) {
	for (j in 1:dim(statobs)[1]) {
		indi=sample(1:nrow(tmp$ss),Nref[i])
		reftable=tmp$ss[indi,]
		param=tmp$param[indi,]
		colnames(reftable) <- c("modindex", colnames(statobs))
		modindex <- as.factor(reftable[1:Nref[i],1])
		sumsta <- reftable[1:Nref[i],-1]
		mc.rf <- abcrf(modindex,sumsta,paral=TRUE,ntrees_in_forest)
		pred.rf <- predict(mc.rf,statobs[j,],paral=TRUE,ntrees_in_forest)
		output <- rbind(output, c(Nref[i], j, as.numeric(pred.rf[1]),
			round(as.numeric(pred.rf[length(pred.rf)]),4),
			unlist(pred.rf[2:(length(pred.rf)-1)]),
			round(as.numeric(mc.rf$prior.err),4)))
	}
}

output <- output[-1,]
```

```
colnames(output) <- c("Nref", "Statobs_row_ID", "Best_scenario", "Posterior_probability",
rownames(as.data.frame(unlist(pred.rf[2:(length(pred.rf)-1)]))), "Prior_error_rate")
write.table(output, output_file_name, quote=FALSE, row.names=FALSE)

# Duration of the analysis (including reading the file reftable.txt)
T2<-Sys.time()
duration = difftime(T2,T1)
duration

rm(list=ls())

# END OF COMPUTATION: Open and edit the output file (output_file_name as defined above) which contains the numerical results of the ABC RF analyses: for each analysis outputs are presented as following: Nref Statobs_row_ID Best_scenario Posterior_probability vote_scen1 vote_scen2 vote_scen3 vote_scen4 Prior_error_rate
```

References cited in Appendix S2

Cornuet JM, Pudlo P, Veyssier J, Dehne-Garcia A, Gautier M, Leblois R, Marin JM, Estoup A. 2014. DIYABC v2.0: a software to make approximate Bayesian computation inferences about population history using single nucleotide polymorphism, DNA sequence and microsatellite data. Bioinformatics. 30, 1187–1189.

Pudlo P, Marin JM, Estoup A, Cornuet JM, Gautier M, Robert CP. 2016. Reliable ABC model choice via random forests. Bioinformatics. 32: 859–866.

R Development Core Team. 2008. R: A language and environment for statistical computing. R Foundation for Statistical Computing, Vienna, Austria. ISBN 3-900051-07-0, URL
